# Equality, diversity and inclusion strategies of NIHR biomedical research centres and clinical research facilities across England: a qualitative content analysis

**DOI:** 10.1136/bmjopen-2025-109321

**Published:** 2026-02-19

**Authors:** Phuong Hua, Shoba Dawson, Hazel Phillips

**Affiliations:** 1 Nuffield Department of Primary Care Health Sciences, University of Oxford, Oxford, UK; 2 Sheffield Centre for Health and Related Research, University of Sheffield, Sheffield, UK; 3 NIHR Bristol Biomedical Research Centre, Bristol, UK

**Keywords:** Education, Medical, Health Education, HEALTH SERVICES ADMINISTRATION & MANAGEMENT, Health Workforce, Health Equity

## Abstract

**Abstract:**

**Objectives:**

The National Institute for Health and Care Research (NIHR) has 20 Biomedical Research Centres (BRCs) and 28 Clinical Research Facilities (CRFs) that work with NHS organisations and universities to translate cutting-edge research into new interventions. As mandated by NIHR, all BRCs/CRFs have an Equality, Diversity and Inclusion (EDI) strategy which details how they will implement EDI through their practices, research and organisational systems. This UK-based study aimed to conduct a pilot qualitative analysis of EDI strategies to compare all 20 NIHR BRCs/CRFs, identify common priorities and improve inclusion across research infrastructures. The analysis was presented at the first in-person seminar for NIHR EDI professionals (Birmingham, October 2024).

**Design:**

Qualitative content analysis of publicly available EDI strategy documents.

**Setting:**

48 research infrastructures (20 BRCs and 28 CRFs).

**Methods:**

EDI strategies were collated into NVivo and Microsoft Excel where inductive coding and content analysis was executed for objectives, action plans and success measures. Both quantitative and qualitative content analyses were conducted to analyse the prevalence of categories and similarities or differences between them. Logic models were developed to map the process of implementing EDI for each main category generated.

**Results:**

The most common main category across objectives was ‘Cultural change in workplaces’ for BRCs and ‘Leadership, governance and policy’ for CRFs. For action plans, codes for ‘Collaborations and Networks’ and ‘Research development and delivery’ were most prevalent for BRCs—for CRFs, it was ‘Workforce culture change’ and ‘Research development and delivery’. Success measures for both BRCs and CRFs most often related to ‘Summary reports, feedback, audits and monitoring’. Differences between BRCs and CRFs reflected their organisational roles and strategic maturity, with BRCs tending to have more comprehensive, measurable strategies.

**Conclusions:**

This study provides the first systematic analysis of EDI strategies across all NIHR BRCs and CRFs, offering a comprehensive mapping of how EDI priorities are articulated and operationalised across objectives, action plans and success measures. While both infrastructures align with NIHR’s inclusion goals, BRCs generally showed more strategic maturity than CRFs. As the analysis was based solely on publicly available strategy documents, it could not determine the extent to which any strategy had been implemented in practice. Future research is needed to examine implementation and impact. The contribution of this work lies in demonstrating systematically and for the first time the ways in which EDI commitments are framed across NIHR infrastructures and their varying levels of depth and maturity. Our findings support the development of more measurable EDI frameworks and highlight opportunities to strengthen inclusion across NIHR-funded research infrastructures.

**STRENGTHS AND LIMITATIONS OF THIS STUDY**

- The study used a rigorous inductive coding approach, supported by NVivo and Microsoft Excel, allowing for detailed categorisation of objectives, action plans and success measures.
- The analyses were cross-checked by a multidisciplinary team with expertise in Equality, Diversity and Inclusion and qualitative methods.
- Logic models were developed to translate qualitative findings into knowledge mobilisation tools for future strategy development and evaluation, including for non-academic stakeholders.
- As a first-of-its-kind document analysis, the study requires future validation and replication; it analysed only publicly available strategies, without input from the National Institute for Health and Care Research workforce or public contributors, and did not capture internal perspectives or implementation progress, which could be addressed in future studies through stakeholder interviews and longitudinal evaluation.

## Background

### Brief context of health inequalities in National Institute for Health and Care Research

 The National Institute for Health and Care Research (NIHR) remains invested in tackling health inequalities across the UK by fostering an inclusive workforce, and directing research funding towards underserved populations.^1^ This comes at a time when we observe ongoing under-representation of diverse populations in research participation,^2^ cultural issues of bullying, harassment and victimisation in the workforce^3^ and inequities in distribution of health resources (funding, workforce and research distribution and training).^4^

Internationally, research funders have developed structured Equality, Diversity and Inclusion (EDI) frameworks that link measurable progress to research excellence. For example, the Canadian Institutes of Health Research (CIHR) and its Tri-Agency partners - NSERC (Natural Sciences and Engineering Research Council of Canada) and SSHRC (Social Sciences and Humanities Research Council of Canada) have implemented a national EDI Action Plan^5^ that integrates inclusive participation, data transparency and accountability within research funding and governance. Similarly, the Fonds de recherche du Québec has produced the Innovative EDI Practices by Granting Agencies framework,^6^ which documents concrete case examples of how granting agencies have reformed peer-review processes, broadened eligibility criteria and incorporated EDI indicators into evaluation and monitoring systems. Such international examples highlight the growing emphasis on embedding EDI at every stage of the research lifecycle, from funding and leadership to research participation. They also provide a useful benchmark for assessing the strategic maturity of NIHR-funded infrastructures such as Biomedical Research Centres (BRCs) and Clinical Research Facilities (CRFs).

and SSHRC) have implemented a national EDI Action Plan^5^ that integrates inclusive participation, data transparency and accountability within research funding and governance. Similarly, the Fonds de recherche du Québec has produced the Innovative EDI Practices by Granting Agencies framework,^6^ which documents concrete case examples of how granting agencies have reformed peer-review processes, broadened eligibility criteria and incorporated EDI indicators into evaluation and monitoring systems. Such international examples highlight the growing emphasis on embedding EDI at every stage of the research lifecycle, from funding and leadership to research participation. They also provide a useful benchmark for assessing the strategic maturity of NIHR-funded infrastructures such as Biomedical Research Centres (BRCs) and Clinical Research Facilities (CRFs).

There are currently 20 NIHR-funded BRCs and 28 CRFs which bridge academic institutions and National Health Service (NHS) trusts to develop cutting-edge treatments, diagnostics and health technologies.^7 8^ BRCs comprise academics and clinicians who manage collaborations between NHS trusts and universities to deliver new treatments, diagnostics and health technologies through translational research.^9^ CRFs are more specialised research groups that require dedicated space and expertise.^7^ Research across BRCs/CRFs cuts across a range of health and disease specialties including genomics, multimorbidity and rare diseases and ultimately improves patient and public experience of health and care systems.

Systemic disparities in research funding, participation and leadership representation have been identified across the UK research infrastructure,^10^ necessitating deliberate action to promote EDI strategies across all BRCs and CRFs. These strategies are focused on research initiatives that would reduce health inequities and promote inclusion across the BRC and CRF workforce, including research and advisory workforces, research participants and public contributors.

### EDI strategies across NIHR infrastructure

The EDI strategies are partly informed by the wider NIHR Research Inclusion Strategy 2022–2027,^4^ which aims to embed inclusion in research design, funding and delivery and organisational culture and systems. This includes diversifying the NIHR workforce, research workforce, research participants and public. The strategy also acknowledges intersectionality and inequalities associated with all protected characteristics mentioned in the Equality Act 2010.^11^ As part of their funding contracts until 2027, all 20 BRCs in England were mandated to submit a formal EDI strategy that was acceptable to the NIHR and outlined objectives, action plans and success measures. Further, on 27 November 2024, NIHR launched a requirement that funding applicants exemplify how their research will address inequalities in health and social care.^12^ This includes embedding inclusion in all stages of the research lifecycle from design to dissemination. Consequently, it is timely to analyse to what extent inclusion is already embedded in research processes and the workforce and set targets for future equality research across NIHR. Likewise, it is not well understood how existing EDI strategies align with national frameworks such as the Equality Act 2010^11^ and NIHR Race Equality Framework^13^ and translate into measurable improvements in research participation and workforce diversity.

The EDI strategies are publicly available, which presents ample opportunity to evaluate how they are devised and implemented across the 20 BRCs and 28 CRFs. There are known benefits of NIHR-specific projects which collect data and evidence on the progress of equity and inclusion work. For instance, the NIHR Diversity Data Report 2022^13^ helped establish a benchmark and diversity targets from which NIHR could measure their progress in diversifying the workforce. The report identified that applicants from ethnic minority groups are less likely to be successful than white applicants. There is also under-representation of disabled applicants, award holders or professional committee members.

### Rationale for qualitative study

To date, there have been few evidence-informed evaluations or analyses of EDI strategies, collectively in the NIHR workforce to assess their effectiveness and impact. Existing research has largely focused on individual case studies^14^ rather than comprehensive cross-institutional analyses. The Public Mental Health Implementation Centre conducted a qualitative study of the Oxford Health BRC EDI strategies.^14^ They identified enablers and barriers to implementation which produced actionable recommendations for NIHR (eg, explaining why protected characteristics data must be collected and commissioning further research). Additional BRCs and the wider NIHR infrastructure would benefit from a combined analysis of the EDI strategies across all 20 BRCs.

NIHR advocates for a learning approach to improving EDI outcomes and without rigorous evaluation, it is unclear whether BRC and CRF EDI strategies have led to meaningful changes in inclusion practices. In line with this, as part of ongoing EDI initiatives at Bristol and Sheffield BRCs, we conducted an exploratory qualitative analysis of EDI strategies across all 20 BRCs and 28 CRFs, identifying common priorities, challenges and opportunities for improvement. There are known differences between the two NIHR infrastructures—BRCs are large-scale partnerships between leading universities and NHS organisations, focusing on translational research with a broad remit.^9^ In contrast, CRFs are purpose-built facilities within NHS hospitals designed to support primarily clinical research studies (eg, experimental medicine trials).^7^ Although CRFs work closely with BRCs, they more often serve as the practical setting for conducting research studies and funding from NIHR is directed towards maintaining high-quality facilities and trial operations. As BRCs are better known for generating research agendas, innovation and translation, their EDI strategies may differ from CRFs. However, there has not been an exploratory analysis of how EDI strategies across BRCs and CRFs contrast or complement each other.

The timeliness of this study is underscored by the inaugural BRC/CRF Inclusion Conference, held in October 2024 in Birmingham, which marked the first national gathering of EDI leads from BRCs and CRFs across the UK. The conference was led by Bristol BRC and provided a platform for EDI professionals to share challenges and best practices for inclusion throughout the research lifecycle. The lead author for this study (PH) presented findings from the first exploratory analysis of EDI strategies across BRCs and CRFs to inform inclusion managers and promote evaluations of existing EDI strategies. This research article provides a comprehensive summary of the research methods, analysis and findings that were presented. It is anticipated to be an important reference for NIHR EDI leads as they adapt to NIHR requirements that make inclusion an explicit condition of funding for domestic programmes.

### Aims and research questions

The aims of this study were: (1) to identify recurring categories and priorities within EDI strategies across all 20 NIHR BRCs and the 28 CRFs and (2) to compare similarities and differences in how these categories were represented across objectives, action plans and success measures.

Through these aims, we can elucidate the joint and unique ways in which these NIHR infrastructures articulate their commitment to EDI throughout their research and organisational practices, and how these strategies address health inequalities and promote inclusion. The following research questions guided our qualitative analysis:

- What are the recurring categories and priorities outlined within EDI strategies for both NIHR BRCs and CRFs?
- What categories or priorities do NIHR BRC and CRF EDI strategies articulate across objectives, action plans and success measures? What are the similarities and differences in these categories/priorities across BRCs and CRFs?
- What opportunities for strengthening the clarity, measurability and accountability of EDI strategies can be identified through a qualitative analysis of objectives, action plans and success measures across BRCs and CRFs?

## Methods

### Qualitative approach and research paradigm

The study was guided by a constructivist paradigm, which recognises that meaning is constructed through social processes and institutional contexts. This was appropriate for the inductive content analysis (ICA) of EDI strategies, allowing the research team to interpret how equality priorities were framed within organisational documents across the NIHR research infrastructure.

### Design and setting

This study was a qualitative analysis, based within the NIHR Bristol BRC (where it was funded). While the research was conducted and funded through the NIHR Bristol BRC, the setting for the data collection encompassed all 20 NIHR BRC and 28 CRFs across England. Analyses were conducted between June and October 2024. The article was written in accordance with the Standards for Reporting Qualitative Research^15^ - see online supplemental table S1.

### Researcher characteristics and reflexivity

The research team comprised individuals with expertise in health inequalities, inclusive research methodologies and research funding and management. Throughout the analysis, the team engaged in reflexive discussions to acknowledge and mitigate potential biases stemming from their prior experiences and perspectives on EDI. This involved regular meetings to critically examine interpretations, challenge assumptions and ensure that the analysis remained grounded in the data.

### Data collection and materials

The lead researcher conducted a systematic search in June 2024 to identify all publicly available EDI strategy documents for NIHR BRCs and CRFs. The official websites of all 20 BRCs and 28 CRFs listed on the NIHR website were searched manually using site search functions and navigation menus. Consistent keywords were applied across sites, including *Equality, Diversity and Inclusion, EDI strategy, EDI action plan, research inclusion* and *EDI report*. All eligible documents were downloaded and archived in PDF format.

Regarding inclusion and exclusion criteria, our dataset comprised all publicly available NIHR BRC and CRF EDI strategy documents in England. Documents were included if they met the following criteria: (1) explicitly identified as an EDI strategy, action plan or comprehensive EDI report and (2) publicly accessible on an official NIHR BRC/CRF or affiliated institutional website.

Documents were excluded if they: (1) were internal-only, non-public or operational documents not intended as formal EDI strategies; (2) focused on equality legislation compliance rather than strategic EDI objectives and (3) were brief statements without substantive strategy content.

14 BRCs had the same EDI strategy as their corresponding CRFs and 16 CRFs had separate EDI strategies: Maudsley, Newcastle, Nottingham, Oxford, Oxford Health, Sheffield, Alder Hey, Guy’s and St Thomas, Lancashire, King’s, St George’s, Norfolk, Liverpool, Newcastle, Royal Free, Royal Surrey.

## Data analysis

Once identified, each document was downloaded, converted to PDF format where necessary, and imported into NVivo V.14 for qualitative content analysis. Table 1 provides a comprehensive list of all NIHR BRCs and CRFs included in the study, indicating the availability of their respective EDI strategies. In addition to NVivo, Microsoft Excel was used to organise and manage the descriptive elements of the analysis. Excel spreadsheets were developed to catalogue each strategy document, track the presence or absence of codes across BRCs and CRFs and calculate the frequency of subcategories and main categories generated during coding. These spreadsheets also allowed comparisons to be mapped and systematic examination of similarities and differences across infrastructures.

**Table 1:** NIHR research centres with EDI strategies across England

Research centre	BRC EDI strategy	CRF EDI strategy (same as BRC)	Separate CRF EDI strategy
Barts	☑	☑	
Birmingham	☑	☑	
Bristol	☑	☑	
Cambridge	☑	☑	
Exeter	☑	☑	
Great Ormond Street Hospital	☑	☑	
Imperial	☑	☑	
Leeds	☑	☑	
Leicester	☑	☑	
Manchester	☑	☑	
Maudsley	☑		☑
Moorfields	☑	☑	
Newcastle	☑		☑
Nottingham	☑		☑
Oxford	☑		☑
Oxford Health	☑		☑
Royal Marsden	☑	☑	
Sheffield	☑		☑
Southampton	☑	☑	
University College London Hospitals	☑	☑	
Alder Hey			☑
Guy’s and St Thomas			☑
Lancashire			☑
King’s			☑
St George’s			☑
Norfolk			☑
Liverpool			☑
Newcastle			☑
Royal Free			☑
Royal Surrey			☑

BRC, Biomedical Research Centre; CRF, Clinical Research Facility; EDI, Equality, Diversity and Inclusion; NIHR, National Institute for Health and Care Research.

Descriptive statistics were run to calculate the prevalence of subcategories and main categories. We then conducted a pilot ICA based on guidelines by Elo and Kyngäs.^16^ ICA was chosen because it enables the systematic organisation and interpretation of textual data when there is limited prior research or theoretical guidance on the phenomenon under study.^16 17^ ICA involves open coding, categorisation and abstraction to derive patterns and categories directly from the data rather than applying predefined frameworks. The method aligns with a constructivist paradigm, which assumes that knowledge and meaning are co-constructed through interpretation and context rather than discovered as objective truths.^18 19^ In this study, the constructivist stance guided the analytic process by recognising that the EDI strategies were developed within social and organisational contexts, reflecting institutional understandings of equality and inclusion within the NIHR research ecosystem.

The lead researcher (PH), under the supervision of coauthors, collated EDI strategies from each BRC/CRF and imported them into NVivo. PH initially conducted open coding to identify relevant EDI concepts. In this study, because the analysis was conducted within a constructivist paradigm, we did not calculate formal inter-rater reliability statistics. Instead, we used the qualitative approach of iterative, consensus-based coding to ensure analytic rigour. The lead researcher conducted initial open coding on a subset of EDI strategies (eg, 10% of the total documents) to develop a preliminary coding framework. This framework, along with coded extracts, was reviewed by the coauthors. Differences in interpretation were discussed collaboratively until consensus was reached and the coding framework was refined through multiple iterations. The iterative process of coding, discussion and refinement of the coding framework continued until all documents were coded and no new categories emerged, ensuring saturation of themes. This process facilitated shared understanding and consistency across coders while remaining aligned with the principles of ICA.

Inductive coding was the most appropriate method as the aim of the study was to identify consistent priorities directly from the EDI strategies and similarities or differences between them, rather than gathering supporting evidence for any particular theory. An initial coding framework was developed based on similar higher-order headings across the EDI strategies: Objectives (goals and targets), Action plans (initiatives implemented to reach goals/targets) and Success measures (evidence/indicators that objectives have been met). Under each heading, PH organised the initial codes into sub-categories which were then collapsed into higher-order/main categories through an iterative process, cross-checked with the wider team (table 2). These steps of coding and categorisation were implemented for EDI strategies across both BRCs and CRFs. Table 3 displays the categorisation matrix developed inductively from the coding process, and sets of main categories (with corresponding subcategories numbered beneath) for BRCs and CRFs across objectives, action plans and success measures. These categories represent the components of the NIHR infrastructure (workforce, research, participants) where EDI principles were explicitly framed within the strategies.

**Table 2:** Objectives, action plans and success measures across BRCs and CRFs

Objectives							
Main category	BRC			CRF		Similarities	
Leadership, governance and policy	Strategically embed EDI in leadership development (eg, accredited courses for underserved groups) to foster structured leadership pipelines.			Embed EDI in operational governance (eg, meeting agendas, SOPs). Focus on tangible policy documents and structural integration.		Both BRCs and CRFs aim to embed EDI into leadership, governance structures and decision-making. They establish EDI steering groups and champions, integrate EDI into policies and promote inclusive leadership development.	
Cultural change in workplaces	Systemically embed EDI strategy and cultural standards. Focus on institutional alignment and maturity models.			Implement training, disability support and antidiscrimination practices. Emphasise awareness and behaviour change at staff level.		Both focus on creating inclusive, equitable and anti-discriminatory workplace cultures. They prioritise staff development, diversity in recruitment and proactive cultural competency.	
Resource development	Develop inclusive funding mechanisms and strategic training initiatives.			Emphasise operational tools (eg, visit templates) and inclusive staff training.		Both invest in developing EDI training resources and inclusive funding mechanisms. They aim for fair access to training and mentorship across all staff.	
Collaborations and partnerships	Foster institutional and strategic health system partnerships.			Focus on community-level engagement and collaborative access to early-phase trials.		Both establish partnerships with institutions, communities and networks to support EDI. Collaboration is a shared strategy to improve research impact and accessibility.	
Student/career development	Target early outreach and career pipeline development (eg, internships, mentorship).			Prioritise equitable access to career progression and diverse hiring practices.		Both support early career researchers, particularly those from underrepresented backgrounds. They create inclusive career pipelines and mentoring structures.	
Evaluations, audits and monitoring	Evaluate strategic EDI impact across research portfolios.			Conduct participant diversity audits and monitor research representation.		Both evaluate EDI progress through data collection, strategy reviews and monitoring mechanisms. Feedback loops and dashboards are used to inform continuous improvement.	
Data collection	Use data for strategic decisions and leadership accountability.			Collect comprehensive demographic data on both patients and workforce.		Both collect and analyse diversity data across workforce, research participation and governance. They aim to align data practices with NIHR standards and use them for benchmarking.	
Research development	Integrate EDI in research design, methodology and dissemination.			Focus on inclusive recruitment, engagement and operational delivery.		Both promote embedding EDI throughout research design and methodology. They focus on increasing participation from underserved groups.	
Communications	Use strategic communication to champion inclusion and representation.			Prioritise accessibility and communication tailored to underserved communities.		Both strive for inclusive and accessible communication strategies. They use websites, social media, newsletters and events to engage diverse audiences.	
ACTION PLANS							
Theme		BRC	CRF				Similarities
Institutional, structural and systemic change		Focus on strategic policy changes, address health inequalities and embed equity into funding processes.	Emphasise operational EDI forums, review policies through Equality Impact Assessments (EIAs) and implement collaborative mechanisms.				Both BRCs and CRFs embed EDI into institutional frameworks, policies and recruitment systems. They use EIAs, engage EDI forums and align with national charters.
Leadership and governance		Embed EDI in board structures, leadership succession planning and community representation.	Operationalise EDI through SOP reviews, introduce CRF-specific leadership training and align policy mechanisms.				Both support inclusive leadership through training, representation and EDI champions. They embed EDI into governance structures and decision-making processes.
Workforce culture change		Develop culture change strategies through inclusive recruitment, bystander programmes and workplace equity policies.	Deliver structured training, monitored compliance and tailored onboarding practices for inclusivity.				Both foster inclusive recruitment, mandatory EDI training and well-being support. They encourage staff engagement through surveys, induction programmes and cultural awareness initiatives.
Representation		Focus on representative governance, diversify recruitment panels and demographic tracking.	Establish formal targets for participant and workforce diversity and promote role models.				Both aim to reflect local and national diversity across workforce, governance and research participation. They increase PPIE diversity and track progress through demographic monitoring.
Collaborations and networks		Lead national forums and build cross-sector partnerships to shape research agendas.	Form internal and local community networks, and convene EDI forums with public contributors.				Both prioritise partnerships with local communities, third sector groups and national stakeholders. They co-create strategies, share best practices and build internal and external EDI networks.
Student and career development		Implement mentorship programmes, develop PhD recruitment strategies and support leadership pathways.	Create mentorship frameworks, open opportunities for non-medical PIs and track staff progression from underrepresented groups.				Both invest in inclusive internships, mentorship and career progression pathways for underrepresented groups. They offer fellowships, summer schools and early career researcher support.
Reviews and evaluations		Conduct audits and strategic evaluations to refine EDI policies and improve inclusion.	Use feedback loops, track data through dashboards and assess EDI maturity models.				Both monitor EDI progress through audits, feedback surveys, impact assessments and strategy reviews. They evaluate EDI in recruitment, research protocols and organisational practices.
Data collection		Prioritise baseline dataset creation, align data standards and address underrepresentation in research participation.	Collect demographic data using NIHR tools, track diversity via dashboards and assess participant characteristics.				Both collect and analyse demographic data on workforce, research participants and leadership. They align data with NIHR standards and use it to inform strategic decisions.
Research development and delivery		Embed EDI across the research lifecycle, focus on health inequalities and advance inclusive methodologies.	Employ targeted recruitment strategies, deliver local community research and use EDI-aligned participant tools.				Both embed EDI in study design and methodology and address underrepresentation in clinical research. They include underserved voices, co-produce studies and engage public contributors.
Communications and publicity		Showcase diverse voices, promote inclusive messaging and share role model stories.	Enhance accessibility, create EDI digital spaces and target outreach to under-represented groups.				Both promote accessible, inclusive communications and showcase EDI through events, websites and social media. They celebrate EDI milestones and ensure representation in outreach.
SUCCESS MEASURES							
Main category		BRC			CRF		Similarities
Leadership and governance		Embed EDI into strategic policies, promote transparency and hold senior leadership accountable.			Focus on localised governance, use structured forums and embed EDI into operational oversight.		Both BRCs and CRFs embed EDI in leadership structures, decision-making and strategic oversight. They establish dedicated roles (eg, EDI champions) and promote inclusive leadership practices.
Resources, training and investment		Lead with tailored EDI training, research toolkits and funding for fellowships.			Implement training delivery and tracking, with emphasis on accessibility and uptake.		Both invest in bespoke EDI training and resource development. They track training uptake and provide EDI-related funding opportunities.
Networks and collaborations		Build national and institutional alliances, and formal partnerships with community advisors.			Emphasise community forums, ambassador engagement and cross-regional CRF alliances.		Both prioritise internal and external EDI partnerships to share learning and co-create best practices. They engage with national networks and local communities to shape inclusive research agendas.
Monitoring, reporting and evaluation		Use audits, data reviews and strategic feedback to evaluate progress and inform policy.			Rely on detailed local documentation, SMART objectives and internal reporting platforms.		Both collect feedback, publish annual reports and use surveys to evaluate EDI progress. They apply learning from events and feedback to shape future activities.
Data collection							Both collect workforce and participant diversity data and use it for strategic monitoring and evaluation. They analyse gaps, benchmark progress and use data to inform action plans.
Research projects and delivery		Integrate EDI into design and data processes with emphasis on leadership-led inclusivity.			Drive inclusivity through recruitment strategies, community engagement and study toolkits.		Both embed EDI in research design, methodologies and team structures. They focus on underserved groups and increase PPIE representation.
Communications and events		Strategically broadcast EDI initiatives, showcase inclusion through digital platforms and reports.			Promote accessibility and multichannel outreach to communities and public audiences.		Both use digital platforms, events and newsletters to promote EDI initiatives and visibility. They aim for accessible, inclusive and representative communication.
Career development opportunities		Provide structured mentoring, leadership training and equitable recruitment pathways.			Monitor inclusivity in training uptake, support diverse leadership and document career progression.		Both provide mentoring, equitable access to training and support for underrepresented staff. They promote inclusive recruitment, leadership progression and structured support.

BRCs, Biomedical Research Centres; CRFs, Clinical Research Facilities; EDI, Equality, Diversity and Inclusion; NIHR, National Institute for Health and Care Research; PPIE, Patient and Public Involvement and Engagement; SOP, Standard Operating Procedures.

**Table 3:** Main categories and sub-categories (numbered) for BRCs and CRFs across objectives, action plans and success measures

	BRC	CRF
Objectives	Main category 1: Leadership, governance and policy Leadership development and support Governance and oversight Representation and decision-making processes EDI governance and policy implementation	Main category 1: Leadership/governance Governance and structural integration of EDI Embedding EDI in research leadership and strategy Embedding data collection in organisational oversight EDI training and transparent training opportunities
	Main category 2: Cultural change in workplaces Defining and standardising workplace culture Embedding equity, diversity and inclusion Workforce development and career progression Recruitment and retention practices	Main category 2: Cultural change in workplaces Inclusion, accessibility and anti-discrimination practices EDI training and awareness
	Main category 3: Resource development Training and professional development Mentoring and career support Inclusive funding practices	Main category 3: Resource development Enhancing research participant experience Improving training approaches
	Main category 4: Collaborations, partnerships General collaboration and stakeholder engagement Health system and local community partnerships	Main category 4: Collaborations/partnerships Networks and external engagement Public and community engagement Regional and national research collaborations Institutional and higher education collaborations Engagement with national and institutional training programmes
	Main category 5: Student and career development Early engagement and outreach for future scientists Career development and support for workforce and students Structured learning and training initiatives	Main category 5: Student/career development Workforce representation and inclusive recruitment Career development and pipeline diversity Supporting professional growth through training
	Main category 6: Evaluations, audits and monitoring Evaluation of EDI impact and processes	Main category 6: Evaluations and audits/monitoring Assessing research participant diversity Tools for measuring and improving equity in research Longitudinal review of inclusion strategies Measuring and monitoring diversity in research participation Reviewing and standardising communication platforms
	Main category 7: Data collection Diversity data collection Workforce and career progression data Data analysis and utilisation	Main category 7: Data collection Diversity monitoring in research and workforce
	Main category 8: Research development Embedding EDI in research design and methodology Increasing diversity in research participation Community-driven and inclusive research engagement Public and patient involvement and engagement	Main category 8: Research development Expanding research in diverse populations Community engagement in research
	Main category 9: Communications Inclusive and representative communication Visibility and representation in public engagement Digital and online communication EDI-centric communication and awareness	Main category 9: Communications Enhancing research communication for accessibility Targeted communication for underserved communities
Action plans	Main category 1: Institutional, structural and systemic change Structural and systemic barriers in institutions Equity, diversity and inclusion policies and frameworks Workforce progression and inclusion Funding equity and inclusion Addressing inequalities in healthcare Economic and structural investment	Main category 1: Institutional, structural, systemic change EDI policy and structural changes Policy and governance for workforce development Research inclusion policy and structural initiatives Research institution and hospital network collaborations Internal institutional EDI collaboration Research network development and operational collaboration
	Main category 2: Leadership and governance Inclusive leadership and governance structures Embedding EDI in governance and decision-making Leadership development and support for underrepresented groups EDI leadership, steering groups and champions	Main category 2: Leadership and governance Committee and board representation Leadership development and training Governance structures and policy implementation Strategy and progress monitoring
	Main category 3: Workforce culture change Inclusive recruitment and retention strategies Training and awareness to embed EDI in workforce culture Workforce support, well-being and equity Fostering an inclusive and engaged workforce Cross-organisation collaboration and knowledge sharing EDI in research leadership and initiatives	Main category 3: Workforce culture change Training and education initiatives Inclusive recruitment and onboarding Organisational culture and inclusion initiatives EDI working groups and forums
	Main category 4: Representation Workforce representation and diversity in research team Representation in governance and decision-making bodies Representation in public and patient involvement Representation in research participation	Main category 4: Representation Equity in research recruitment and participant diversity Diversity in leadership and workforce
	Main category 5: Collaborations and networks Partnerships with communities, stakeholders and organisations National and local EDI networks and strategic partnerships Internal networks, working groups and collaboration forums Cross-institutional collaboration and knowing sharing Networking and collaboration opportunities	Main category 5: Collaborations and networks National and local EDI networking and partnerships Community engagement and third-sector partnerships Youth and volunteer engagement in research
	Main category 6: Student and career development Career pathways and progression in biomedical research Inclusive recruitment and development of PhD students Internships, fellowships and work experience Mentorship and leadership development Funding opportunities and support for career development Opportunities for leadership and professional engagement	Main category 6: Student/career development Mentorship and career development opportunities Researcher development and support
	Main category 7: Reviews and evaluations Monitoring and evaluation of EDI progress Staff experience, workforce and organisational reviews Research and funding review processes Equality impact Gov and data monitoring Governance and strategic oversight	Main category 7: Reviews and evaluations Evaluation of PPIE activities Accessibility and communication reviews Organisational monitoring and EDI strategy evaluation Data monitoring, gap analysis and metrics development Evaluation of training and career progression Reporting and review processes for EDI activities
	Main category 8: Data collection Standardisation and alignment of data collection systems Collection and monitoring of diversity data Data collection on research participants and underrepresentation Tools and systems for data collection	Main category 8: Data collection Collection and analysis of diversity data Structural and governance-driven data collection Data collection for EDI training and awareness Intersectional and localised data analysis
	Main category 9: Research development and delivery Embedding EDI in research design and methodology Addressing underrepresentation in research participation Public and patient involvement and engagement Research priorities and thematic focus areas Research training, leadership and workforce development Enhancing research infrastructure and delivery Support and resources for researchers Economic and societal impact of research	Main category 9: Research development and delivery Inclusive research recruitment and participation Study design and research infrastructure Community-engaged research delivery PPIE in research design Diversity and inclusion in research participation Evaluation and monitoring of participatory research initiatives
	Main category 10: Communications and publicity Strategic communications planning and working groups Publicity and promotion of EDI initiatives Events, outreach and community engagement Digital and online visibility Dissemination of research and best practices	Main category 10: Communications and publicity Public awareness and engagement initiatives Website and digital accessibility Inclusive research communication and reporting Internal knowledge sharing and staff engagement
Success measures	Main category 1: Leadership and governance Leadership structures and roles Embedding EDI in governance and decision-making Governance meetings and oversight Leadership commitment and accountability	
	Main category 2: Resources/training/investment EDI training and capacity building Development of EDI resources Funding and investment in EDI initiatives	
	Main category eme 3: Networks/collaborations National and institutional collaborations Community engagement and outreach networks Stakeholder and partner engagement	Main category 1: Networks/collaborations Staff networks and internal collaboration External partnerships and stakeholder engagement Community engagement and outreach
	Main category 4: Summary reports/reviews, feedback, audits and monitoring Annual and institutional reports Feedback collection and evaluation Qualitative feedback from events and engagement activities Workforce and demographic data monitoring Auditing and reviewing organisational processes Monitoring research participation and engagement Training and capacity building audits Data-driven decision-making and impact assessment Governance and oversight	Main category 2: Reviews/feedback/audits/monitoring Diversity and equality monitoring Reports on EDI progress and implementation Meeting minutes and monitoring processes Surveys and feedback analysis Monitoring and evaluation of training efforts
	Main category 5: Research projects and delivery Embedding EDI in research design and delivery Patient and public involvement and engagement Researcher support and development Research project and data infrastructure	Main category 3: Research projects and delivery Study design and participant inclusion Community-informed research strategy
	Main category 6: Communications and events EDI communication strategies and plans Website and digital publications Traditional and non-traditional publications Newsletters and media outreach Events and public engagement	Main category 4: Communications and events Digital communication and website content Events and public engagement Internal communication and reporting
	Main category 7: Career development opportunities Mentoring and support structures Training and development programmes Equitable access to career opportunities Recruitment and selection processes	Main category 5: Career and staff development Staff training and capacity building Inclusion and cultural awareness Leadership and researcher development Conference presentations and knowledge dissemination

BRCs, biomedical research centres; CRFs, clinical research facilities; EDI, Equality, Diversity and Inclusion.

## Results

### Quantitative content analysis of EDI strategies for BRCs/CRFs

Descriptive statistics were used to calculate the frequency/prevalence of each main category across the BRCs and CRFs, as specified in table 4. Prevalence was determined by the number of BRC/CRFs for which codes were generated for that main category. For instance, codes for main category 1 (Leadership, governance and policy) were generated for 8 BRCs (40%) and 10 CRFs (35.7%). We have omitted names of specific BRCs/CRFs in the manuscript as it was beyond the scope of the study to conduct in-depth analyses of specific BRCs/CRFs.

**Table 4:** Prevalence of main categories across BRCs/CRFs—objectives, action plans and success measures

Objectives	BRCs/20 N (%)	CRFs/28 N (%)	Action plans	BRCs/20 N (%)	CRFs/28 N (%)	Success measures	BRCs/20 N (%)	CRFs/28 N (%)
Main category 1: Leadership, governance and policy	8 (40)	10 (35.7)	Main category 1: Institutional, structural and systemic change	11 (55)	13 (46.4)	Main category 1: Leadership and governance	12 (60)	0
Main category 2: Cultural change in workplaces	18 (90)	7 (25)	Main category 2: Leadership and governance	15 (75)	9 (32.1)	Main category 2: Resources/training/investment	9 (45)	0
Main category 3: Resource development	9 (45)	5 (17.9)	Main category 3: Workforce culture change	16 (80)	14 (50)	Main category 3: Networks/collaborations	12 (60)	8 (28.6)
Main category 4: Collaborations, partnerships	8 (40)	8 (28.6)	Main category 4: Representation	10 (50)	7 (25)	Main category 4: Summary reports/reviews, feedback, audits and monitoring	12 (60)	13 (46.4)
Main category 5: Student and career development	6 (30)	7 (25)	Main category 5: Collaborations and networks	17 (85)	10 (35.7)	Main category 5: Research projects and delivery	8 (40)	6 (21.4)
Main category 6: Evaluations, audits and monitoring	6 (30)	8 (28.6)	Main category 6: Student and career development	14 (70)	4 (14.3)	Main category 6: Communications and events	10 (50)	7 (25)
Main category 7: Data collection	7 (35)	4 (14.3)	Main category 7: Reviews and evaluations	16 (80)	14 (50)	Main category 7: Career development opportunities	7 (35)	8 (28.6)
Main category 8: Research development	15 (75)	6 (21.4)	Main category 8: Data collection	15 (75)	10 (35.7)			
Main category 9: Communications	6 (30)	5 (17.9)	Main category 9: Research development and delivery	17 (85)	14 (50)			
			Main category 10: Communications and publicity	16 (80)	9 (32.1)			

BRCs, biomedical research centres; CRFs, clinical research facilities.

### Qualitative content analysis of EDI strategies BRCs/CRFs

In addition to quantitative analyses, we conducted a qualitative and descriptive interpretation of the supporting evidence under each category to explore dominant patterns in EDI priorities. These steps were implemented to analyse all higher-order headings of the EDI strategies: Objectives, Action plans and Success Measures. Although we generated similar categories for objectives, action plans and success measures across the BRCs and CRFs, there were subtle qualitative differences in their priorities and focus, some of which are summarised in online supplemental table S2. The main qualitative differences are discussed below in relation to six main categories.

#### Evaluations/Audits

BRCs tended to focus on strategic oversight and long-term evaluation. Guy’s and St Thomas’ (GST) BRC committed to an “*annual review of our progress and the adaptation of our projects and actions to increase our momentum of EDI maturation*.” Evaluation often included structural oversight such as “*case-study libraries*” and “*impact assessments of anonymised panels*.” Success was measured via metrics on recruitment, training uptake and governance diversity.

CRFs emphasised operational evaluations, especially in research participation and focused on improving equity in research. Oxford Health CRF noted the need to “*review the CRF research participant EDI monitoring questionnaire,*” while Alder Hey CRF planned to “*audit study participation to determine where inequalities in participation lie*”. Success was measured by survey feedback, review of CRF recruitment portfolios and EDI maturity reporting.

#### Student/career development

BRCs showcased structured educational and career initiatives and promoted early engagement and outreach for future scientists. Exeter BRC promoted “*outreach activities to engage young people in biomedical science*” and Nottingham BRC established a “*Summer School programme*” and Patient and Public Involvement and Engagement (PPIE)-related projects for trainees. BRCs prioritised long-term capacity building via internships, mentorships and pipeline programmes (eg, Oxford, Nottingham, Maudsley), with clear links to leadership development and PhD recruitment from under-represented groups. BRCs tracked success via mentorship structures, equitable access to fellowships and career progression monitoring—often linked to broader institutional benchmarks (eg, Imperial’s Springboard programme, Exeter’s internship support, GOSH’s training schemes).

CRFs primarily focused on broadening representation and equitable access and recruitment. St George’s CRF intends its workforce to be “*a similar reflection of the Trust workforce and local community ensuring EDI representation, especially in senior levels*,” while GST CRF expressed interest in hosting an “*international nurse*” as part of The International Association of Clinical Research Nurses programme. CRFs also concentrated on workforce readiness and structured onboarding, including inclusive job descriptions, leadership training frameworks (Royal Free) and mentorship schemes for clinical research (Royal Free). CRFs assessed impact through targeted recruitment and leadership progression—for example, tracking studies led by non-medical or early career PIs (Oxford, Alder Hey), monitoring diversity in grant applicants, and success stories from training initiatives (eg, Lancashire’s intern project).

#### Collaborations

BRCs prioritised high-level stakeholder and policy engagement. Moorfields and Royal Marsden BRCs focused on working with stakeholders ‘for impact and sustainability,’ while Nottingham BRC highlighted its efforts to partner with Integrated Care System structures. There were action plans to frequently convene national forums and share best practices through networks (eg, Oxford, Bristol, Birmingham). BRCs defined success by their leadership role in shaping the national agenda (eg, Leicester), creation of shared EDI resources (eg, Manchester’s case study library), and formation of institutional partnerships (eg, Royal Marsden’s best practice network).

CRFs tended to highlight grassroots and operational partnerships (people on committees and advisory boards, research networks). Liverpool CRF formed a “*working group with patient and public contributors from underrepresented groups*,” and Royal Free CRF outlined establishing partnerships “*with health/referral services working in women’s, LGBTQ+, youth, aged care, disability, or Black and minority ethnic health*.” A notable difference is CRFs’ explicit mention of regional and national research collaborations, including to “*Work with other regional CRFs, and paediatric CRFs nationally, to establish other EDI work that has had a positive and negative impact (Years 3–5) (Alder Hey*)”. CRFs demonstrated success through formalised partnerships (eg, Memorandum of Understanding, MOU in Royal Free), active involvement in CRF alliances (eg, Alder Hey), and community engagement events (eg, Africa Oye).

#### Leadership/governance

BRCs focused on EDI in governance and decision-making. Manchester BRC aimed to establish an ‘EDI Steering Group with representatives from diverse communities,’ and both Barts and Exeter BRC will initiate ‘EDI check-in sessions with the management board. GOSH BRC planned to ‘review the composition of key boards and decision-making committees’. BRC success measures included EDI champions across themes (eg, Oxford, GOSH), recording and analysing governance meeting minutes (eg, Imperial), and holding leadership to account via compliance audits (eg, UCLH’s ‘comply or explain’ policy).

Similarly, CRFs emphasised structural integration and accountability. Imperial CRF included ‘EDI Champions on each Theme Board, in the BRC Executive and the CRF Management Committee.’ Regular progress monitoring was part of governance agendas (eg, Alder Hey), and there was a strong focus on policy structure (eg, Standard Operating Procedures (SOPs), MOU development). Success measures for CRFs focused on formal reporting through CRF Operational Groups (eg, Liverpool), integration of EDI into SOPs (Lancashire) and appointment of inclusive leadership roles with clear monitoring expectations (eg, King’s, Royal Marsden).

#### Research development/delivery

BRCs integrated EDI at all stages of research, from concept and design to dissemination. Manchester BRC aimed to ‘embed and promote EDI projects into the core business,’ and UCLH BRC is building a ‘database of individuals from underserved groups interested in participating in trials.’ Initiatives such as Sheffield’s NIHR INCLUDE-based assessments aimed to address structural barriers to recruitment. Success measures for BRCs included long-term integration of EDI in research planning, for example, inclusive design frameworks (Oxford, Sheffield), evidence of EDI-informed grant applications (Royal Marsden), and PPIE embedded in thematic strategies (Cambridge).

CRFs implemented inclusion practices at the delivery level. Nottingham CRF adopted a ‘standardised approach to translation and alternative formats,’ and Alder Hey CRF developed a ‘checklist to consider EDI-related issues in study design.’ CRFs also highlighted community engagement in research, with objectives like ‘Provide support for at least one collaborative community engagement project per year (Oxford Health)’. Nottingham deployed mobile research units and creative community outreach. Success measures for CRFs were delivery-oriented, such as increased diversity of study participants (eg, Alder Hey), new tools to support inclusive recruitment (eg, Liverpool’s EDI toolkit), and integration of EDI in community-informed study review groups (eg, King’s, Royal Free).

#### Communications

BRCs focused on representative, high-level communications and visibility. Exeter BRC ensures ‘communications are representative of our diverse workforce, alongside ensuring relevant opportunities are promoted to a wide-ranging audience’ while UCLH BRC included EDI at ‘research symposia and showcase events.’ Action plans included EDI newsletters (Manchester), website accessibility audits (Oxford), diversity in public-facing content (Exeter) and filming of EDI champions (Imperial). BRCs measured success via public visibility: active websites, publication of EDI reports and dashboards (eg, Imperial, UCLH), digital newsletters, and showcasing inclusive research practices online (eg, Oxford Health).

CRFs centred on accessibility and community engagement. Alder Hey CRF sought the best formats to ‘present information about studies to families where English proficiency may be a barrier.’ Initiatives included review of CRF website readability with PPIE input (Sheffield), codesigned communications with local groups (Oxford Health), and digital engagement that surpassed NHS communication standards (Nottingham). CRFs measured success based on communication accessibility and outreach: ensuring website readability (eg, King’s, Oxford Health), regular updates on EDI progress, community event participation (eg, Liverpool) and internal staff engagement via forums and newsletters (eg, Lancashire, GST).

## Common main categories across objectives, action plans and success measures for BRCs/CRFs

To examine the consistency of priorities throughout EDI planning, we identified which main categories were represented across all components of EDI strategies: objectives, action plans and success measures. This analysis generated six main categories which are showcased in online supplemental table S3), accompanied by a description and examples (textual excerpts from EDI strategies) of objectives, action plans and success measures across the BRC/CRFs.

BRCs generally had higher thematic prevalence overall across categories, despite being fewer in number. CRFs typically have less developed EDI strategies with fewer metrics and less breadth across the main categories. They consistently scored less than 50% prevalence on most categories across objectives, action plans and success measures.

Through the iterative process of coding and categorisation, we synthesised the relationships between objectives, action plans and success measures into novel logic models, providing a visual representation of the EDI planning process for each main category. These logic models depict the main stages of EDI planning and the questions and actions that could be undertaken at each stage to achieve success measures. The section below provides a detailed description of two logic models for the main categories that were showcased at the inclusion conference. All figures are provided as separate files.

**Figure 1:**
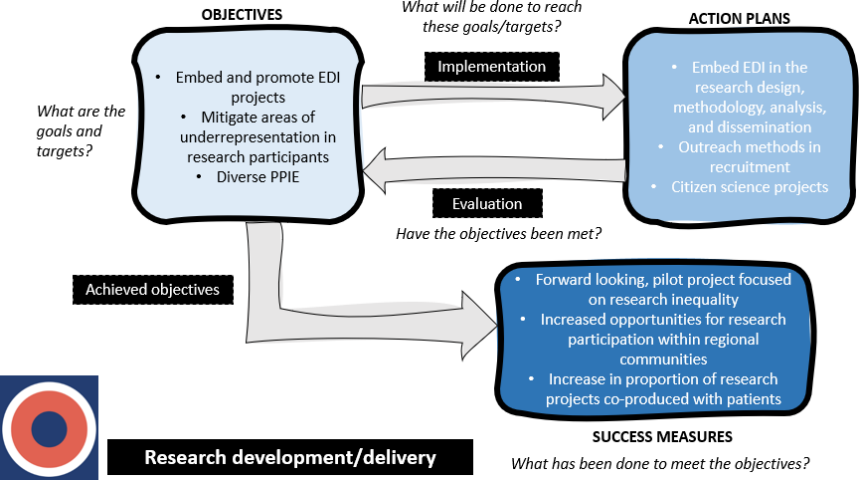
This logic model depicts the processes involved in delivering on objectives related to Research development/delivery, based on the EDI strategies. EDI, Equality, Diversity and Inclusion; PPIE, Patient and Public Involvement and Engagement.

Figure 1 depicts the processes involved in delivering on objectives related to Research development. It also shows the prompts/questions at each stage that BRCs/CRFs may benefit from discussing. Beginning with objectives, BRCs/CRFs must decide on their goals/targets. Some specific goals from the collective BRC/CRF strategies are listed there (eg, designing and promoting EDI projects). Before devising action plans, BRCs/CRFs could discuss implementation (what will be done to reach these goals/targets). Some specific action plans are listed (eg, embedding EDI in research design and methodology). Then, BRCs/CRFs could conduct an evaluation of whether the objectives have been met. If they have achieved their objectives, they should observe success measures (eg, the development of a pilot project focused on research inequality).

**Figure 2:**
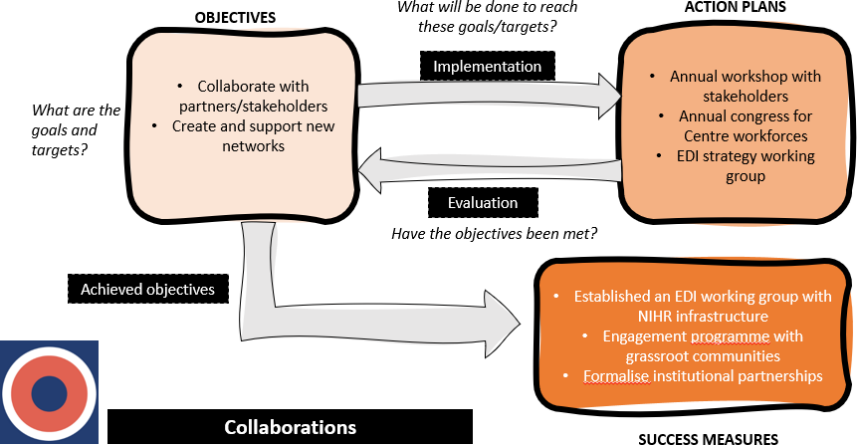
This logic model depicts the processes involved in delivering on objectives related to Collaborations, based on the EDI strategies. EDI, Equality, Diversity and Inclusion; NIHR, National Institute for Health and Care Research.

Figure 2 depicts the processes involved in delivering on objectives related to Collaborations. Beginning with objectives, BRCs/CRFs must plan to collaborate with partners/stakeholders and cultivate new networks. Specific action plans include annual workshops, congresses and EDI working groups. Success measures from these action plans may include an established working group, engagement programmes with grassroot communities and institutional partnerships.

**Figure 3:**
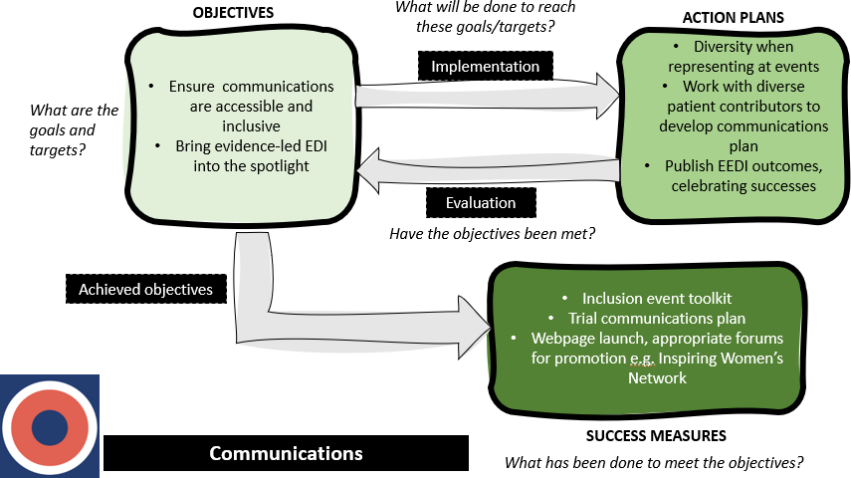
This logic model depicts the processes involved in delivering on objectives related to Communications, based on the EDI strategies. EDI, Equality, Diversity and Inclusion.

Figure 3 depicts the processes involved in delivering on objectives related to Communications. Beginning with objectives, BRCs/CRFs aim to ensure communications are accessible and inclusive and to bring evidence-led EDI into the spotlight. Specific action plans include ensuring diversity when representing at events, working with diverse patient contributors to develop communication plans and publishing EDI outcomes to celebrate successes. Success measures from these actions may include an inclusion event toolkit, a trial communication plan and the launch of webpages.

Online supplemental figure S1-S3 are included in the supplementary materials and are logic models for the remaining categories: leadership/governance (online supplemental figure S1), evaluations/audits (online supplemental figure S2) and student/career development (online supplemental figure S3). Similar to figures1^3^, the logic models display objectives, action plans and success measures with prompts/questions at each stage that BRCs/CRFs may benefit from discussing.

## Discussion

The findings reveal considerable variability in depth, maturity and measurability of EDI implementation. To address the original aim and research questions, this pilot study successfully mapped EDI priorities across all 20 BRCs and 28 CRFs. We identified six key main categories or priorities for EDI intervention, that were recurring throughout objectives, action plans and success measures–Evaluations/audits, Student and career development, Collaborations, Leadership/governance, Research development/delivery and Communications. The only exception was that Leadership/governance was not a main category for success measures of CRFs, perhaps pointing to gaps in accountability structures. These categories were most prevalent in the action plans of EDI strategies, particularly Collaborations and Research Development/delivery for BRCs and Evaluations/audits and Research Development/delivery for CRFs. These findings suggest that while both BRCs and CRFs share common EDI priorities, their strategic emphasis differs. BRCs appear to focus more on fostering collaborative environments and enhancing research development and delivery, reflecting their broader translational research remit.^9^ In contrast, CRFs place greater emphasis on evaluation mechanisms and the delivery of research, indicating a more operational and performance-driven approach to EDI.^7^

Overall, the results indicate that while NIHR-mandated EDI strategies provide a foundation for inclusion across research and workforce systems, there is variability in strategy depth, implementation planning and evaluation metrics and further development is required to achieve long-term, measurable progress. Noticeably, there are different priorities at each stage. For BRCs, cultural change in the workplace dominated the objectives, while collaborations and research development featured most in action plans, and leadership/governance in success measures. CRFs, by contrast, emphasised collaborations and audits in objectives, cultural change and research delivery in action plans, and audits in success measures. This may reflect imbalances or biases in how EDI strategies are written or actual disparities in what BRC/CRFs prioritise at each stage of EDI planning. The observation that success measures often linked to monitoring and reporting for both BRCs and CRFs reveals a tendency towards metrics as a measure of impact.

There could also be efforts to differentiate between how action plans and success measures are outlined in EDI strategies. For instance, under the main category of Student/career development, the action plans include Mentorship, doctoral opportunities and internships. Success measures tended to replicate these plans and instead, could include distinct measurable outcomes of success such as data that records improvements in uptake of these opportunities. This is where the logic models developed in this study could be implemented to better delineate objectives, action plans and success measures more explicitly. To deploy these EDI strategies, there continues to be heavy reliance on the NIHR workforce and increased involvement of research participants and the public could maximise inclusion. This is pertinent in the context of NIHR’s successful co-production work with public contributors to establish the Race Equality Framework.^20^ Some success measures across the main categories set specific achievements and measurable indicators including named EDI coordinators or champions, policy, bespoke training and funding schemes. However, we cannot determine from these EDI strategies what impacts these success measures have had and whether they were achieved (ie, names of policies and training programmes). Statements from each research facility about specific milestones and evaluation metrics or standardised benchmarks for evaluating EDI progress could be provided in future EDI annual reports to ensure greater accountability. Likewise, some success measures reported in the strategies (eg, toolkits, communication plans, webpages) reflect outputs rather than evaluative indicators. This signifies a tendency across BRCs/CRFs to name deliverables as indicators of strategic progress and success (rather than evaluation metrics).

### Comparison of BRCs and CRFs: variability in strategy maturity and scope

The BRCs and CRFs share similar priorities across the objectives, action plans and success measures of their EDI strategies. This is perhaps explained by their complementary roles within the NIHR infrastructure that requires alignment on core EDI principles for consistency. There is also a clear commitment to inclusive governance across both BRCs and CRFs, EDI in workforce training and recruitment and use of audits and feedback loops to evaluate EDI progress. At the same time, the leading categories across objectives, action plans and success measures were generally not the same for BRCs and CRFs, suggesting different strategic priorities. The exceptions were that for action plans across both BRCs and CRFs, research development and delivery featured among the most prevalent main categories. For success measures, audits/monitoring emerged as a leading category across both BRCs and CRFs.

Key differences between the two infrastructures suggest BRCs generally present more comprehensive and strategic documents whereas CRFs have less developed EDI strategies with fewer metrics and less breadth across the main categories, reflecting differences in resources and roles. BRCs had higher thematic prevalence overall, despite being fewer in number. CRFs consistently scored less than 50% prevalence on most categories across objectives, action plans and success measures. There were subtle qualitative differences in priorities between the infrastructures—BRCs tended to emphasise strategic planning (eg, leadership pipelines) whereas CRFs focused on practical implementation (eg, changes to SOPs, onboarding processes). Importantly, BRCs tended to integrate cultural change into institutional alignment and had clearer success measures linked to strategic outcomes. CRFs commonly framed cultural change around individual behaviour and relied on activity reporting (eg, events, local feedback). These disparities may reflect differing organisational remits and resources given that BRCs generate EDI research and agenda-setting whereas CRFs are designed to support the implementation of the research.

### Strengths and weaknesses of the study

This study has several methodological strengths. It is the first cross-institutional qualitative content analysis of publicly available EDI strategies across all NIHR BRC and CRFs in England, offering a comprehensive view of the national research infrastructure. The analysis followed established qualitative content analysis guidelines and was grounded in a systematic, inductive coding approach using NVivo and Excel. The research process was further strengthened by collaborative cross-checking among a multidisciplinary team with expertise in EDI and qualitative research. Additionally, the development of logic models from the qualitative data provides a novel and practical framework that can guide future EDI strategy planning, implementation and evaluation across similar institutional contexts.

However, the analysis was the first of its kind and requires validation and replication in future studies. Future evaluations could involve the NIHR workforce, public contributors and advisory groups like the NHS Race and Health Observatory. There could be ongoing funding to invest in charting the progress and impact of EDI strategies as the BRCs/CRFs continue to evolve. We acknowledge that each EDI strategy is at different stages of maturity and future research could incorporate insights that are not publicly available (eg, from interviews with EDI leads about actual implementation and operationalisation across their BRC/CRF).

### Strengths and weaknesses in relation to other studies

There are no readily available prior studies that have undertaken a similar systematic textual analysis of these specific NIHR policy documents, reinforcing the originality of our study. Previous studies within the NIHR infrastructure have primarily focused on single-site case studies or evaluations of specific frameworks such as the NIHR Race Equality Framework.^20^ For example, Papageorgiou *et al*^14^ conducted a qualitative mapping and scoping project at the Oxford Health BRC to explore enablers and barriers to implementing EDI strategies. Despite the absence of direct comparative content analyses, the study’s findings can be meaningfully contextualised by comparing them with the overarching NIHR Research Inclusion Strategy and related initiatives. This provides a crucial lens for understanding the consistency and coherence of EDI efforts across the NIHR ecosystem. The six main categories identified in this study—Research development and delivery, Collaborations, Communications, Leadership/governance, Evaluations/audits and Student/career development—are highly aligned with the NIHR Research Inclusion Strategy 2022–2027. This national strategy prioritises inclusion across the entire research lifecycle, promoting leadership accountability, improving data and evaluation, enhancing inclusive research design and fostering workforce diversity. For example, the study’s focus on research development and collaboration echoes the NIHR strategy’s emphasis on inclusive practices in research funding and delivery. Similarly, the inclusion of leadership and governance, as well as evaluations and audits, aligns with NIHR’s commitment to systems-level accountability and continuous improvement. The attention to communications and career development reflects NIHR’s strategic goal to build an inclusive culture and enable progression for under-represented groups across the research workforce. This alignment reinforces the relevance of the categories developed through this content analysis for guiding future implementation and evaluation of NIHR’s inclusion strategy.

### Implications for NIHR and EDI leads and stakeholders

The study addresses a highly pertinent and critical area within UK health research, aligning with significant national priorities. The findings point to a need for standardised evaluation criteria and reporting templates that clearly distinguish objectives, action plans and success indicators. This aligns with the broader direction of NIHR policy, which continues to introduce mandates for inclusion strategies that will transform how studies and resources are commissioned as well as policy frameworks. For instance, as of Autumn 2024, NIHR required all applicants for domestic programme awards to detail how they will ensure inclusion is embedded into the entire research process. This funding condition mandates that inclusion must be costed and justified throughout the application, affecting how studies are designed, commissioned and resourced. This mandate highlights a growing operational and strategic need for a tiered EDI strategy framework with baseline requirements, followed by context-specific recommendations for BRCs and CRFs. There could also be greater interinstitutional collaboration to codesign strategies and share resources across BRCs and CRFs that are colocated.

We acknowledge that concepts identified in our main categories, such as research development, collaborations and governance, are widely recognised within organisational planning and evaluation. However, the contribution of our analysis lies not in naming these concepts, but in demonstrating systematically and for the first time how they are articulated and operationalised across all NIHR BRC and CRF EDI strategies. This article also provides a pilot mapping of how each category spans objectives, action plans and success measures, and their depth and maturity across BRCs and CRFs. Studies such as ours prepare the NIHR workforce, including directors, NHS and University partners, researchers and policy makers, for delivering on EDI and their targets and refining national inclusion mandates, strategy guidance and evaluation frameworks. EDI leads across BRCs/CRFs now have more benchmarking resources (eg, logic models) to support planning and monitoring of local EDI strategies. Future research can replicate our study methodology and integrate an analysis of enablers and barriers to implementation, including demographic, institutional and funding contexts, bringing together the NIHR workforce and associate partners, similar to a previous study.^14^ This can be done closely for individual BRC/CRFs as well as collectively to facilitate comparisons. Regular seminars or conferences that unify the NIHR workforce are ideal platforms to showcase findings from EDI evaluations and monitor implementation. Although the strategy documents provide insight into objectives, action plans and intended success measures, they do not reveal whether initiatives have been implemented as planned, nor whether they have led to measurable changes. Further comparative evaluations of EDI strategies could assess the effectiveness or maturity of implementation. They could also analyse the consistency of these strategies with the wider NIHR strategy to identify broader commonalities, unique challenges and best practices in EDI strategic approaches across diverse contexts.

We address the possibility that there are widely known strategies or approaches (eg, systems-level equity frameworks, equity-centred evaluation models, organisational change frameworks, structural accountability mechanisms) that were not consistently observed across BRCs and CRFs. Their absence in the data does not imply that they are not being used within NIHR infrastructures, only that they were not present in publicised strategies.

This study was based solely on publicly available strategy documents which did not provide an assessment of the progress made on individual actions or objectives. Likewise, we could not determine the extent to which any strategy had been implemented in practice. It is also notable that the term ‘embedded’ was used within the EDI strategies to describe intentions or aspirations, yet the documents did not provide sufficient detail to evaluate whether such aspirations had translated into operational change. We additionally acknowledge that this term appeared in our coding framework as part of the naming of subcategories. Taking into account the reviewer’s feedback, this terminology was not the most appropriate descriptor for what could be evidenced from the data, and we recognise this as a limitation of the analysis. Future research would be required to understand implementation and progress, including approaches such as interviews with EDI leads, case studies of how strategies are operationalised and evaluation using agreed indicators. These studies can provide a greater understanding of whether and how EDI strategies are truly ‘embedded’ within NIHR infrastructures.

While the present study focuses on NIHR-funded BRCs and CRFs in England, it offers insights that are transferable to other national research systems seeking to operationalise and evaluate EDI frameworks within multi-institutional infrastructures. Together, these international models (eg, CIHR EDI) and the NIHR EDI frameworks highlight a global movement towards funder-led accountability and data-driven inclusion in health research.

## Conclusions

This pilot study is the first cross-infrastructure analysis of EDI strategies in NIHR BRCs/CRFs and coincided with the first in-person seminar of EDI leads. The findings demonstrate that while NIHR’s inclusion mandates have fostered a common foundation, variability remains in how objectives, action plans and success measures are articulated and operationalised. The novel logic models provide a practical visual tool for improving measurability and accountability in future NIHR EDI planning. In the next decade, we can expect that this evidence base will pave the way for longitudinal and implementation studies which will improve NIHR equality frameworks, public action groups and inclusive health and care research. This will have transformative effects for greater diversity in research participation, leading to more inclusive treatments, diagnostics and medical technologies and positive outcomes for underserved populations.

## Supplementary material

10.1136/bmjopen-2025-109321 online supplemental file 1

## Data Availability

Data are available on reasonable request.
