# Supplementary material for: Equality, diversity and inclusion strategies of NIHR biomedical research centres and clinical research facilities across England: a qualitative content analysis
Source: BMJ Open. 2026 Feb 19;16(2):e109321. doi: 10.1136/bmjopen-2025-109321 (PMC12927318; doi:10.1136/bmjopen-2025-109321)
Supplement: online supplemental file 1 [file bmjopen-16-2-s001.docx]

# Supplementary data

## Figure S1


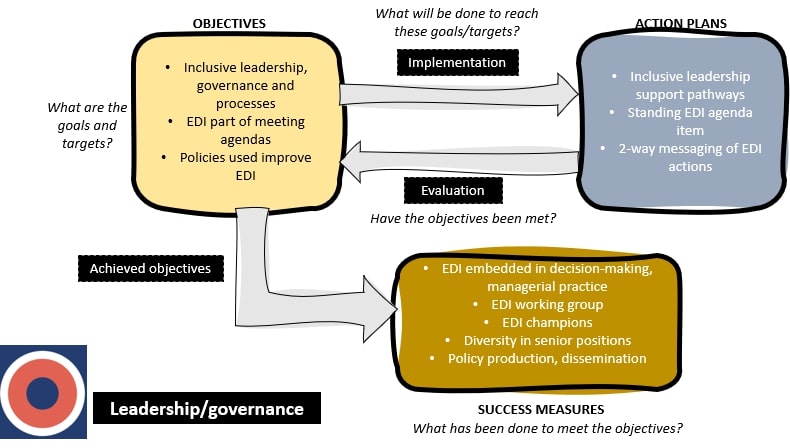


**Figure S1. This logic model depicts the processes involved in delivering on objectives related to Leadership/governance, based on the EDI strategies.**

## Figure S2


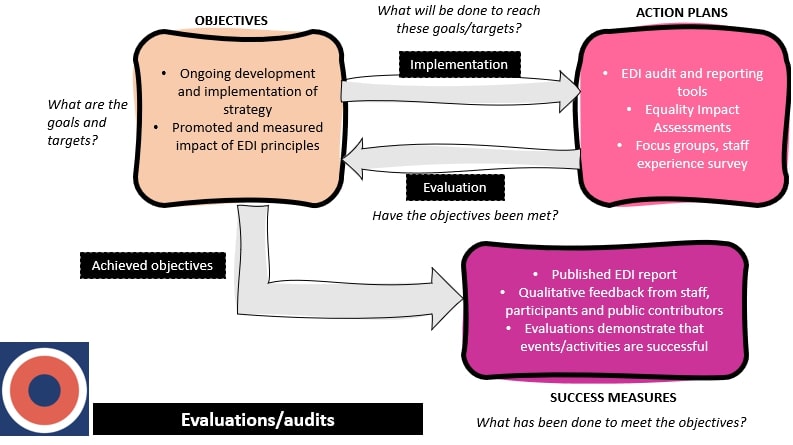


**Figure S2. This logic model depicts the processes involved in delivering on objectives related to Evaluations/audits, based on the EDI strategies.**

## Figure S3


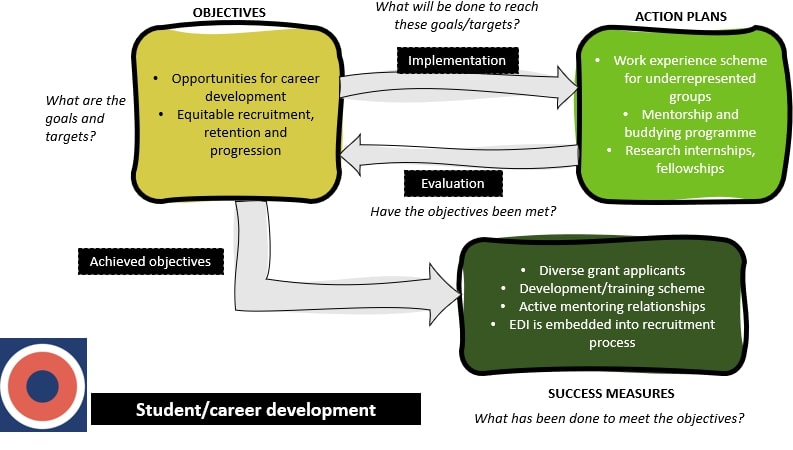


**Figure S3. This logic model depicts the processes involved in delivering on objectives related to Student/career development, based on the EDI strategies.**

# **Table S1. Standards for Reporting Qualitative Research (SRQR)**

| **Title and abstract** |  |
| --- | --- |
| Title | The title identifies the study as a qualitative content analysis. |
| Abstract | The structured abstract includes objectives, design, setting, methods, results, and conclusions. |
| **Introduction** |  |
| Problem formulation | The background section describes the need to understand how EDI strategies are developed and implemented across BRCs and CRFs and why this is important for health equity. |
| Purpose/research question | Aims are clearly stated towards the end of the introduction. Research questions are explicitly listed. |
| **Methods** |  |
| Qualitative approach and research paradigm | Described in the methodology as an inductive content analysis of public documents using NVivo, grounded in qualitative descriptive principles. The study was guided by a constructivist paradigm to recognise that meaning is constructed through social processes and institutional contexts. This paradigm also aligns with the study’s aim to understand the EDI strategies rather than to test hypotheses or generalise statistically. |
| Researcher characteristics and reflexivity | Roles and expertise of the research team are outlined in the author contributions and acknowledgements. The lead author is a research fellow with expertise in health inequalities. She has led research at the intersection of systemic inequalities, racial justice, and health service delivery, including national evaluations of institutional racism in NHS inpatient mental health care. Her experience includes co-producing research with underserved populations and integrating EDI principles into study design, implementation, and knowledge mobilisation.  Dr Shoba Dawson is a senior research fellow in Inclusive Research and has led initiatives to increase diversity in clinical trials and health research through strategic partnerships with community organisations across the UK.  Dr Hazel Phillips is the Chief Operating Officer of the NIHR Bristol Biomedical Research Centre, with a professional background in research funding, governance, and operational leadership. She has contributed to multiple institutional initiatives aimed at improving equality, diversity and inclusion (EDI) across health research infrastructures. Her work has supported the development and implementation of inclusive research governance frameworks and organisational strategies. |
| Context | The manuscript explains the institutional context (NIHR BRCs and CRFs in England) and provides rationale. |
| Sampling strategy | All publicly available EDI strategies from all 20 BRCs and 28 CRFs were included — total population sample. Rationale for inclusion criteria is explained. |
| Ethical issues pertaining to human subjects | Ethics approval and consent addressed under declarations. No ethics approval was required for document analysis. |
| Data collection methods | Sources of documents, extraction into NVivo/Excel, and coding procedures are described. Iterative development of categories is noted. |
| Data collection instruments and technologies | NVivo and Excel were used for coding and organising content. |
| Units of study | Defined as 48 strategy documents from 20 BRCs and 28 CRFs. Level of inclusion is detailed. |
| Data processing | Data entry, coding processes and validation are described in the methods. |
| Data analysis | Inductive content analysis based on Elo & Kyngäs (2008) is cited. Analysis conducted iteratively and collaboratively by a qualitative team. |
| Techniques to enhance trustworthiness | Cross-checking by the two co-authors of the study with expertise in EDI strategies and use of logic models are described. To enhance trustworthiness, a subset of EDI strategy documents was independently analysed by the other co-authors. Initial coding discrepancies were identified and discussed collaboratively, with consensus reached through iterative team discussions. This process ensured consistency in code application and strengthened the credibility of the inductive content analysis.  The research team actively reflected on their positionality and potential influence on data interpretation. Researchers brought different disciplinary backgrounds and lived experiences relevant to EDI, which informed a critical and reflexive approach to analysis. Reflexive discussions were held throughout the coding and interpretation phases to consider how researcher assumptions might shape the findings. |
| **Results/findings** |  |
| Synthesis and interpretation | Key themes and main categories are described with synthesis across the NIHR infrastructure. |
| Links to empirical data | Descriptive tables and category definitions support findings. Supplementary materials (e.g. logic models) substantiate analysis. |
| **Discussion** |  |
| Integration with prior work, implications, transferability, and contribution(s) to the field | The discussion connects findings to NIHR policy documents (e.g., Inclusion Strategy 2022–2027) and includes implications for multiple stakeholders. This study provides the first comprehensive qualitative content analysis of EDI strategies across all NIHR BRCs and CRFs, thereby filling a significant gap in the literature. It offers an overview of how a major UK health research infrastructure formally articulates its commitment to EDI at the institutional level. |
| Limitations | Described in both strengths and limitations sections. Includes reflection on scope (publicly available data), absence of stakeholder interviews, and need for replication. |
| **Other** |  |
| Conflicts of interest | Stated in the ‘Competing interests’ section before the Background. |
| Funding | Clearly reported in ‘Funding statement’ before the Background. |

# **Table S2. Main categories and sub-categories (numbered) for BRCs and CRFs across objectives, action plans and success measures**

|  | **BRC** | **CRF** |
| --- | --- | --- |
| **Objectives** | Main category 1: Leadership, governance and policy   1. Leadership development and support 2. Governance and oversight 3. Representation and decision-making processes 4. EDI governance and policy implementation | Main category 1: Leadership/governance   1. Governance and structural integration of EDI 2. Embedding EDI in research leadership and strategy 3. Embedding data collection in organizational oversight 4. EDI training and transparent training opportunities |
|  | Main category 2: Cultural change in workplaces   1. Defining and standardizing workplace culture 2. Embedding equity, diversity and inclusion 3. Workforce development and career progression 4. Recruitment and retention practices | Main category 2: Cultural change in workplaces   1. Inclusion, accessibility and anti-discrimination practices 2. EDI training and awareness |
|  | Main category 3: Resource development   1. Training and professional development 2. Mentoring and career support 3. Inclusive funding practices | Main category 3: Resource development   1. Enhancing research participant experience 2. Improving training approaches |
|  | Main category 4: Collaborations, partnerships   1. General collaboration and stakeholder engagement 2. Health system and local community partnerships | Main category 4: Collaborations/partnerships   1. Networks and external engagement 2. Public and community engagement 3. Regional and national research collaborations 4. Institutional and higher education collaborations 5. Engagement with national and institutional training programs |
|  | Main category 5: Student and career development   1. Early engagement and outreach for future scientists 2. Career development and support for workforce and students 3. Structured learning and training initiatives | Main category 5: Student/career development   1. Workforce representation and inclusive recruitment 2. Career development and pipeline diversity 3. Supporting professional growth through training |
|  | Main category 6: Evaluations, audits and monitoring   1. Evaluation of EDI impact and processes | Main category 6: Evaluations and audits/monitoring   1. Assessing research participant diversity 2. Tools for measuring and improving equity in research 3. Longitudinal review of inclusion strategies 4. Measuring and monitoring diversity in research participation 5. Reviewing and standardizing communication platforms |
|  | Main category 7: Data collection   1. Diversity data collection 2. Workforce and career progression data 3. Data analysis and utilisation | Main category 7: Data collection   1. Diversity monitoring in research and workforce |
|  | Main category 8: Research development   1. Embedding EDI in research design and methodology 2. Increasing diversity in research participation 3. Community-driven and inclusive research engagement 4. Public and patient involvement and engagement | Main category 8: Research development   1. Expanding research in diverse populations 2. Community engagement in research |
|  | Main category 9: Communications   1. Inclusive and representative communication 2. Visibility and representation in public engagement 3. Digital and online communication 4. EDI-centric communication and awareness | Main category 9: Communications   1. Enhancing research communication for accessibility 2. Targeted communication for underserved communities |
| **Action plans** | Main category 1: Institutional, structural and systemic change   1. Structural and systemic barriers in institutions 2. Equity, diversity and inclusion policies and frameworks 3. Workforce progression and inclusion 4. Funding equity and inclusion 5. Addressing inequalities in healthcare 6. Economic and structural investment | Main category 1: Institutional, structural, systemic change   1. EDI policy and structural changes 2. Policy and governance for workforce development 3. Research inclusion policy and structural initiatives 4. Research institution and hospital network collaborations 5. Internal institutional EDI collaboration 6. Research network development and operational collaboration |
|  | Main category 2: Leadership and governance   1. Inclusive leadership and governance structures 2. Embedding EDI in governance and decision-making 3. Leadership development and support for underrepresented groups 4. EDI leadership, steering groups and champions | Main category 2: Leadership and governance   1. Committee and board representation 2. Leadership development and training 3. Governance structures and policy implementation 4. Strategy and progress monitoring |
|  | Main category 3: Workforce culture change   1. Inclusive recruitment and retention strategies 2. Training and awareness to embed EDI in workforce culture 3. Workforce support, wellbeing and equity 4. Fostering an inclusive and engaged workforce 5. Cross-organisation collaboration and knowledge sharing 6. EDI in research leadership and initiatives | Main category 3: Workforce culture change   1. Training and education initiatives 2. Inclusive recruitment and onboarding 3. Organisational culture and inclusion initiatives 4. EDI working groups and forums |
|  | Main category 4: Representation   1. Workforce representation and diversity in research team 2. Representation in governance and decision-making bodies 3. Representation in public and patient involvement 4. Representation in research participation | Main category 4: Representation   1. Equity in research recruitment and participant diversity 2. Diversity in leadership and workforce |
|  | Main category 5: Collaborations and networks   1. Partnerships with communities, stakeholders and organisations 2. National and local EDI networks and strategic partnerships 3. Internal networks, working groups and collaboration forums 4. Cross-institutional collaboration and knowing sharing 5. Networking and collaboration opportunities | Main category 5: Collaborations and networks   1. National and local EDI networking and partnerships 2. Community engagement and third-sector partnerships 3. Youth and volunteer engagement in research |
|  | Main category 6: Student and career development   1. Career pathways and progression in biomedical research 2. Inclusive recruitment and development of PhD students 3. Internships, fellowships and work experience 4. Mentorship and leadership development 5. Funding opportunities and support for career development 6. Opportunities for leadership and professional engagement | Main category 6: Student/career development   1. Mentorship and career development opportunities 2. Researcher development and support |
|  | Main category 7: Reviews and evaluations   1. Monitoring and evaluation of EDI progress 2. Staff experience, workforce and organizational reviews 3. Research and funding review processes 4. Equality impact Gov and data monitoring 5. Governance and strategic oversight | Main category 7: Reviews and evaluations   1. Evaluation of PPIE activities 2. Accessibility and communication reviews 3. Organisational monitoring and EDI strategy evaluation 4. Data monitoring, gap analysis and metrics development 5. Evaluation of training and career progression 6. Reporting and review processes for EDI activities |
|  | Main category 8: Data collection   1. Standardisation and alignment of data collection systems 2. Collection and monitoring of diversity data 3. Data collection on research participants and underrepresentation 4. Tools and systems for data collection | Main category 8: Data collection   1. Collection and analysis of diversity data 2. Structural and governance-driven data collection 3. Data collection for EDI training and awareness 4. Intersectional and localized data analysis |
|  | Main category 9: Research development and delivery   1. Embedding EDI in research design and methodology 2. Addressing underrepresentation in research participation 3. Public and patient involvement and engagement 4. Research priorities and thematic focus areas 5. Research training, leadership and workforce development 6. Enhancing research infrastructure and delivery 7. Support and resources for researchers 8. Economic and societal impact of research | Main category 9: Research development and delivery   1. Inclusive research recruitment and participation 2. Study design and research infrastructure 3. Community-engaged research delivery 4. PPIE in research design 5. Diversity and inclusion in research participation 6. Evaluation and monitoring of participatory research initiatives |
|  | Main category 10: Communications and publicity   1. Strategic communications planning and working groups 2. Publicity and promotion of EDI initiatives 3. Events, outreach and community engagement 4. Digital and online visibility 5. Dissemination of research and best practices | Main category 10: Communications and publicity   1. Public awareness and engagement initiatives 2. Website and digital accessibility 3. Inclusive research communication and reporting 4. Internal knowledge sharing and staff engagement |
| **Success measures** | Main category 1: Leadership and governance   1. Leadership structures and roles 2. Embedding EDI in governance and decision-making 3. Governance meetings and oversight 4. Leadership commitment and accountability |  |
|  | Main category 2: Resources/training/investment   1. EDI training and capacity building 2. Development of EDI resources 3. Funding and investment in EDI initiatives |  |
|  | Main category eme 3: Networks/collaborations   1. National and institutional collaborations 2. Community engagement and outreach networks 3. Stakeholder and partner engagement | Main category 1: Networks/collaborations   1. Staff networks and internal collaboration 2. External partnerships and stakeholder engagement 3. Community engagement and outreach |
|  | Main category 4: Summary reports/reviews, feedback, audits and monitoring   1. Annual and institutional reports 2. Feedback collection and evaluation 3. Qualitative feedback from events and engagement activities 4. Workforce and demographic data monitoring 5. Auditing and reviewing organizational processes 6. Monitoring research participation and engagement 7. Training and capacity building audits 8. Data-driven decision-making and impact assessment 9. Governance and oversight | Main category 2: Reviews/feedback/audits/monitoring   1. Diversity and equality monitoring 2. Reports on EDI progress and implementation 3. Meeting minutes and monitoring processes 4. Surveys and feedback analysis 5. Monitoring and evaluation of training efforts |
|  | Main category 5: Research projects and delivery   1. Embedding EDI in research design and delivery 2. Patient and public involvement and engagement 3. Researcher support and development 4. Research project and data infrastructure | Main category 3: Research projects and delivery   1. Study design and participant inclusion 2. Community-informed research strategy |
|  | Main category 6: Communications and events   1. EDI communication strategies and plans 2. Website and digital publications 3. Traditional and non-traditional publications 4. Newsletters and media outreach 5. Events and public engagement | Main category 4: Communications and events   1. Digital communication and website content 2. Events and public engagement 3. Internal communication and reporting |
|  | Main category 7: Career development opportunities   1. Mentoring and support structures 2. Training and development programmes 3. Equitable access to career opportunities 4. Recruitment and selection processes | Main category 5: Career and staff development   1. Staff training and capacity building 2. Inclusion and cultural awareness 3. Leadership and researcher development 4. Conference presentations and knowledge dissemination |

# **Table S3. Objectives, action plans and success measures across BRCs and CRFs**

| **OBJECTIVES** | | | | | | | |
| --- | --- | --- | --- | --- | --- | --- | --- |
| **Main category** | **BRC** | | | **CRF** | | **Similarities** | |
| Leadership, governance and policy | Strategically embed EDI in leadership development (e.g., accredited courses for underserved groups) to foster structured leadership pipelines. | | | Embed EDI in operational governance (e.g., meeting agendas, SOPs). Focus on tangible policy documents and structural integration. | | Both BRCs and CRFs aim to embed EDI into leadership, governance structures, and decision-making.  They establish EDI steering groups and champions, integrate EDI into policies, and promote inclusive leadership development. | |
| Cultural change in workplaces | Systemically embed EDI strategy and cultural standards.  Focus on institutional alignment and maturity models. | | | Implement training, disability support, and anti-discrimination practices.  Emphasize awareness and behaviour change at staff level. | | Both focus on creating inclusive, equitable, and anti-discriminatory workplace cultures.  They prioritise staff development, diversity in recruitment, and proactive cultural competency. | |
| Resource development | Develop inclusive funding mechanisms and strategic training initiatives. | | | Emphasise operational tools (e.g., visit templates) and inclusive staff training. | | Both invest in developing EDI training resources and inclusive funding mechanisms.  They aim for fair access to training and mentorship across all staff. | |
| Collaborations and partnerships | Foster institutional and strategic health system partnerships. | | | Focus on community-level engagement and collaborative access to early-phase trials. | | Both establish partnerships with institutions, communities, and networks to support EDI.  Collaboration is a shared strategy to improve research impact and accessibility. | |
| Student/career development | Target early outreach and career pipeline development (e.g., internships, mentorship). | | | Prioritise equitable access to career progression and diverse hiring practices. | | Both support early career researchers, particularly those from underrepresented backgrounds.  They create inclusive career pipelines and mentoring structures. | |
| Evaluations, audits and monitoring | Evaluate strategic EDI impact across research portfolios. | | | Conduct participant diversity audits and monitor research representation. | | Both evaluate EDI progress through data collection, strategy reviews, and monitoring mechanisms.  Feedback loops and dashboards are used to inform continuous improvement. | |
| Data collection | Use data for strategic decisions and leadership accountability. | | | Collect comprehensive demographic data on both patients and workforce. | | Both collect and analyse diversity data across workforce, research participation, and governance.  They aim to align data practices with NIHR standards and use them for benchmarking. | |
| Research development | Integrate EDI in research design, methodology, and dissemination. | | | Focus on inclusive recruitment, engagement, and operational delivery. | | Both promote embedding EDI throughout research design and methodology.  They focus on increasing participation from underserved groups. | |
| Communications | Use strategic communication to champion inclusion and representation. | | | Prioritise accessibility and communication tailored to underserved communities. | | Both strive for inclusive and accessible communication strategies.  They use websites, social media, newsletters, and events to engage diverse audiences. | |
| **ACTION PLANS** | | | | | | | |
| **Theme** | | **BRC** | **CRF** | | | | **Similarities** |
| **Institutional, structural and systemic change** | | Focus on strategic policy changes, address health inequalities, and embed equity into funding processes. | Emphasise operational EDI forums, review policies through Equality Impact Assessments (EIAs), and implement collaborative mechanisms. | | | | Both BRCs and CRFs embed EDI into institutional frameworks, policies, and recruitment systems.  They use Equality Impact Assessments (EIAs), engage EDI forums, and align with national charters. |
| **Leadership and governance** | | Embed EDI in board structures, leadership succession planning, and community representation. | Operationalise EDI through Standard Operating Procedures (SOP) reviews, introduce CRF-specific leadership training, and align policy mechanisms. | | | | Both support inclusive leadership through training, representation, and EDI champions.  They embed EDI into governance structures and decision-making processes. |
| **Workforce culture change** | | Develop culture change strategies through inclusive recruitment, bystander programmes, and workplace equity policies. | Deliver structured training, monitored compliance, and tailored onboarding practices for inclusivity. | | | | Both foster inclusive recruitment, mandatory EDI training, and wellbeing support.  They encourage staff engagement through surveys, induction programs, and cultural awareness initiatives. |
| **Representation** | | Focus on representative governance, diversify recruitment panels, and demographic tracking. | Establish formal targets for participant and workforce diversity and promote role models. | | | | Both aim to reflect local and national diversity across workforce, governance, and research participation.  They increase PPIE diversity and track progress through demographic monitoring. |
| **Collaborations and networks** | | Lead national forums and build cross-sector partnerships to shape research agendas. | Form internal and local community networks, and convene EDI forums with public contributors. | | | | Both prioritise partnerships with local communities, third sector groups, and national stakeholders.  They co-create strategies, share best practices, and build internal and external EDI networks. |
| **Student and career development** | | Implement mentorship programmes, develop PhD recruitment strategies, and support leadership pathways. | Create mentorship frameworks, open opportunities for non-medical PIs, and track staff progression from underrepresented groups. | | | | Both invest in inclusive internships, mentorship, and career progression pathways for underrepresented groups.  They offer fellowships, summer schools, and early career researcher support. |
| **Reviews and evaluations** | | Conduct audits and strategic evaluations to refine EDI policies and improve inclusion. | Use feedback loops, track data through dashboards, and assess EDI maturity models. | | | | Both monitor EDI progress through audits, feedback surveys, impact assessments, and strategy reviews.  They evaluate EDI in recruitment, research protocols, and organisational practices. |
| **Data collection** | | Prioritise baseline dataset creation, align data standards, and address underrepresentation in research participation. | Collect demographic data using NIHR tools, track diversity via dashboards, and assess participant characteristics. | | | | Both collect and analyse demographic data on workforce, research participants, and leadership.  They align data with NIHR standards and use it to inform strategic decisions. |
| **Research development and delivery** | | Embed EDI across the research lifecycle, focus on health inequalities, and advance inclusive methodologies. | Employ targeted recruitment strategies, deliver local community research, and use EDI-aligned participant tools. | | | | Both embed EDI in study design and methodology and address underrepresentation in clinical research.  They include underserved voices, co-produce studies, and engage public contributors. |
| **Communications and publicity** | | Showcase diverse voices, promote inclusive messaging, and share role model stories. | Enhance accessibility, create EDI digital spaces, and target outreach to underrepresented groups. | | | | Both promote accessible, inclusive communications and showcase EDI through events, websites, and social media.  They celebrate EDI milestones and ensure representation in outreach. |
| **SUCCESS MEASURES** | | | | | | | |
| **Main category** | | **BRC** | | | **CRF** | | **Similarities** |
| **Leadership and governance** | | Embed EDI into strategic policies, promote transparency, and hold senior leadership accountable. | | | Focus on localised governance, use structured forums and embed EDI into operational oversight. | | Both BRCs and CRFs embed EDI in leadership structures, decision-making, and strategic oversight.  They establish dedicated roles (e.g. EDI champions) and promote inclusive leadership practices. |
| **Resources, training and investment** | | Lead with tailored EDI training, research toolkits, and funding for fellowships. | | | Implement training delivery and tracking, with emphasis on accessibility and uptake. | | Both invest in bespoke EDI training and resource development.  They track training uptake and provide EDI-related funding opportunities. |
| **Networks and collaborations** | | Build national and institutional alliances, and formal partnerships with community advisors. | | | Emphasise community forums, ambassador engagement, and cross-regional CRF alliances. | | Both prioritise internal and external EDI partnerships to share learning and co-create best practices.  They engage with national networks and local communities to shape inclusive research agendas. |
| **Monitoring, reporting and evaluation** | | Use audits, data reviews, and strategic feedback to evaluate progress and inform policy. | | | Rely on detailed local documentation, SMART objectives, and internal reporting platforms. | | Both collect feedback, publish annual reports, and use surveys to evaluate EDI progress.  They apply learning from events and feedback to shape future activities. |
| **Data collection** | |  | | |  | | Both collect workforce and participant diversity data and use it for strategic monitoring and evaluation.  They analyse gaps, benchmark progress, and use data to inform action plans. |
| **Research projects and delivery** | | Integrate EDI into design and data processes with emphasis on leadership-led inclusivity. | | | Drive inclusivity through recruitment strategies, community engagement, and study toolkits. | | Both embed EDI in research design, methodologies, and team structures.  They focus on underserved groups and increase PPIE representation. |
| **Communications and events** | | Strategically broadcast EDI initiatives, showcase inclusion through digital platforms and reports. | | | Promote accessibility and multi-channel outreach to communities and public audiences. | | Both use digital platforms, events, and newsletters to promote EDI initiatives and visibility.  They aim for accessible, inclusive, and representative communication. |
| **Career development opportunities** | | Provide structured mentoring, leadership training, and equitable recruitment pathways. | | | Monitor inclusivity in training uptake, support diverse leadership, and document career progression. | | Both provide mentoring, equitable access to training, and support for underrepresented staff.  They promote inclusive recruitment, leadership progression, and structured support. |

# **Table S4. Prevalence of six main categories and examples of objectives, action plans and success measures**

|  | **BRCs** | | | **CRFs** | | |  | | | |
| --- | --- | --- | --- | --- | --- | --- | --- | --- | --- | --- |
| **Main category** | **Objectives**  **N(%)** | **Action plans**  **N(%)** | **Success Measures**  **N(%)** | **Objectives**  **N(%)** | **Action plans**  **N(%)** | **Success Measures**  **N(%)** | **Description** | **Objectives** | **Action plans** | **Success measures** |
| Evaluations/audits | 6(30) | 16(80) | 12(60) | 8 (28.6) | 14(50) | 13(46.4) | Ongoing development, implementation and evaluation of EDI strategy | *Promote and measure impact of EDI principles across the BRC research portfolio and our research teams* | *Monitor outcomes from the NHS Workforce Disability Equality Standard (WRES) and the Workforce Race Equality Standard (WRES) and incorporate recommendations* | *Production of data collection template to monitor workforce equality standards* |
| Student/career development | 6(30) | 14(70) | 7(35) | 7(25) | 4(14.3) | 8(28.6) | Career opportunities and development for future generations, junior workforce and underrepresented groups | *Build upon already established partnerships and form new ones to secure programmes/funds to enable young people from underserved groups to undertake internships and work experience placements* | *Pilot approaches for addressing disparities in (funding) applications from certain groups* | *Robust and equitable recruitment process for all career stages, using best practices from other institutions/NIHR infrastructures* |
| Collaborations | 8(40) | 17(85) | 12(60) | 8(28.6) | 10(35.7) | 8(28.6) | Collaborations and networks to improve training, interventions, research and EDI activities | *Develop EDI activities with partners and stakeholders* | *Work alongside partners to monitor workforce equality data* | *Established an EDI working group with other NIHR infrastructure* |
| Leadership/governance | 8(40) | 15(75) | 12(60) | 10(35.7) | 9(32.1) | 0(0) | EDI embedded in leadership positions, committees and boards ensuring representation, oversight and governance | *EDI Champions to sit on each Main category Board, in the BRC Executive and the CRF Management Committee* | *Review composition of our key boards and decision-making committees* | *Equality, Diversity, and Inclusion are truly embedded into all areas and considered in decision-making, teaching, managerial practice and in student engagement* |
| Research development/delivery | 15(75) | 17(85) | 8(40) | 6(21.4) | 14(50) | 6(21.4) | Promote EDI throughout the research process and involve underrepresented groups in research design and delivery | *Mitigate areas of underrepresentation in our research participants* | *Set up a project to explore and address any underrepresentation in new research studies or clinical trials* | *Completed a paper on outreach with underserved communities* |
| Communications | 6(30) | 16(80) | 10(50) | 5(17.9) | 9(32.1) | 7(25) | Representative and inclusive communications including events, presentations and web sources | *Ensure communications are accessible and understandable* | *Engage with our communities and underserved populations through events, social media and co-designed webinars* | *We will have developed an evidence-led EDI case study library that highlights EDI work* |
