# Supplementary material for: Equality, diversity and inclusion strategies of NIHR biomedical research centres and clinical research facilities across England: a qualitative content analysis
Source: BMJ Open. 2026 Feb 19;16(2):e109321. doi: 10.1136/bmjopen-2025-109321 (PMC12927318; doi:10.1136/bmjopen-2025-109321)
Supplement: Supplementary file 2 [file bmjopen-2025-109321.reviewer_comments.pdf]

PEER REVIEW HISTORY

BMJ Open publishes all reviews undertaken for accepted manuscripts. Reviewers are asked to complete a checklist review form and are provided with free text boxes to elaborate on their assessment. These free text comments are reproduced below.

ARTICLE DETAILS

Title (Provisional)

Equality, Diversity and Inclusion Strategies of NIHR Biomedical Research Centres and Clinical Research Facilities across England: A Qualitative Content Analysis

Authors

Hua, Phuong; Dawson, Shoba; Phillips, Hazel

VERSION 1 - REVIEW

**Reviewer**	**1**
**Name**	**Bradley, Elizabeth**
**Affiliation**	**Yale University, Vassar College**
**Date**	**28-Sep-2025**
**COI**	**None**

Very timely, very thorough - I like the paper very much. The graphics add much and I would just footnote them in terms of the sources for the data and the overall meaning so they can stand alone. Well done!

**Reviewer**	**2**
**Name**	**Sim, Meaghan**
**Affiliation**	**IWK Health Centre**
**Date**	**10-Nov-2025**
**COI**	**None**

Thank you for the opportunity to review this manuscript. I have organized comments based on the rubric outlined by the journal.

1. **Originality** - does the work add enough to what is already in the published literature? If so, what does it add?

This is an interesting topic and of importance to the research enterprise. While I appreciate the work is located within the UK – I would encourage the authors to expand

the relevance of this work and describe additional work that may have been done globally that explore EDI frameworks and their outcomes, within research enterprises in similar health systems. This could easily be added to the background and then referred again within the discussion. For example, the Canadian Institutes of Health Research has an EDI strategy and framework where progress is reported. See: Equity, Diversity and Inclusion (EDI) in Action at CIHR - CIHR (see page 2, lines 49-50, rationale for work). See also: Fonds de recherche du Québec (FRQ) Innovative-EDI-Practices-by-Granting-Agencies.pdf which describes EDI practices using case examples.

2. **Importance of work to readers** - does this work matter to researchers, clinicians, patients, teachers, or policymakers?

As previously stated, this work is timely and important to the research enterprise and decision-makers as the research community aims to embed EDI within their practices. See previous above to expand relevance of the work to a more global audience and situate this work within a global perspective – as written, including within the discussion, it is very focused on NIHR and relevance within the UK.

3. **Evidence-based medicine** - is the evidence presented scientifically and

reliable? Please see proceeding comments.

4. **Research question** - is it clearly defined and appropriately answered?
5. **Study design** - is the overall design adequate?

While the research question(s) are provided, I have some comments on these. Design is appropriate to address at least the first two research objectives: 1) What are the recurring categories and priorities outlined within EDI strategies for both NIHR BRCs and CRFs? and
2) What are the similarities and differences in the stated objectives, action plans, and success measures within the EDI strategies of NIHR BRCs and CRFs? I am unclear about objective 3 in relation to the design: "What recommendations can be made to enhance the strategic development, measurability and accountability of EDI strategies within NIHR- funded research infrastructure?" (p.4, lines 95 to 97); whether the design of the study allows for this objective to be measured. The authors note that they have only relied on publicly available documents for analyses and a limitation is the lack of partner engagement to understand the nuances and implementation considerations surrounding these strategies. Unless these documents specifically address recommendations within their evaluation(s), the ability to answer this objective would be limited within current approach/project scope.

6. **Participants** - are they adequately described and their conditions well defined?

The units of analyses for this study are the documents/strategies within each BRC and

CRF.

7. **Methods** - are they adequately described? Are they ethical?

The authors situate this work within a constructivist paradigm using inductive content analyses. While the methods are appropriate, please add additional detail on ICA for the reader and supporting citations. Constructivist paradigm should also be referenced. (see p. 10, lines 100-104). This research used publicly available documents; no human ethics required. However, did this study undergo an ethics exemption within the sponsoring institution? Please confirm and state the same or provide proof of exemption (will defer to BMJ editor for this). Please confirm that data management guidelines were followed based on NIHR Bristol Biomedical Research Centre institutional guidelines.

Search and Analysis: p. 11 – lines 125-27 – who is we? Were websites searched by 1 author or multiple authors? Was a search approach used for website search? For example, how many webpages were followed? Were Boolean operators and or specific phrases applied to each webpage? What was searched for? More details on the systematic approach would be helpful. Further, if there were multiple reviewers, was there a pilot and then an IRR conducted? Please explain. Were there specific inclusion/exclusion criteria? Mention this. There are a noted lack of details in the methods.

Line 127 – the sentence that starts with "some were written" – please clarify what "some" is – does this refer to the strategies? Lines 128 to 130 also read like findings.

P. 12 – lines 147 to 149 – how was reliability of coding assessed among multiple reviewers? Or was there only one coder for all documents? Please clarify.

There seem to be some inconsistencies with what is written in the methods, and the detail/information provided on the SRQR and in the abstract. The comments made in relation to the SRQR should be embedded within the narrative of the manuscript. For example, Excel is mentioned as an organization tool for the analysis (also in abstract) but how it's used is not described within the methods section.

8. **Results** - do they answer the research question? Are they credible and well- presented?

The authors organize their findings narratively and in table format, with logic models developed. Would the authors consider organizing their findings in alignment with their study objectives? This would help with reader interpretation. At this stage, and with the evidence they have collected, I don't know if the separate logic models contribute anything further to the findings and detract from the purpose of this work. Why have separate logic models for each category? Pages 13 to 14 describe well known approaches to conducting planning and evaluation work within service-oriented

organizations and do not provide novel contributions to the literature. In another example, success measures are presented as outputs rather than evaluation metrics – e.g., toolkits, communications plans, and webpages.

9. **Interpretation and conclusions** - are they warranted by and sufficiently derived from/focused on the data? Is the message clear?

The message isn't as clear as it could be and I think this has to do with the organization of the findings/results as well as the depth of reflections found within the discussion section. The authors should be commended for conducting this work – there is a clear need to understand how research-supporting entities like BRCs and CRFs can integrate EDI into their practices. While the authors do discuss implementation in their discussion, I would encourage them to consider further the limitations of what they have observed (see: lines 361-363, lack of detail on what's been achieved and why some strategies may or may not be successful). Are we at risk of finding success in low-hanging strategies where the more important strategies – governance, leadership, policy may be more impactful? Moreover, are there strategies that they didn't observe, that the literature suggests could be important?

I'm also focusing on the authors use of "embedded" in relation to the identified strategies (p. 12, lines 161-162). My interpretation of the aim of this work is to categorize the EDI strategies as they've been named or identified within these NIHR entities – linking to objective 1, and to describe similarities and differences between the BRCs and CRFs – addressing objective 2. From the detail provided further along in the authors' analysis, the existing level of operationalization or "embeddedness" is open to interpretation. For example, evidence of evaluation findings varies across the documents reviewed and how success is defined varies according to the strategy and/or action. Based on this – is there even an understanding of the progress made on each of these actions/objectives? Of note is that some outputs are named but few details provided. I'm not clear that the evidence within this study can speak (at this point) to the level of embeddedness of EDI strategies or not. This is one of the inherent challenges of EDI work – it is well-meaning, needed/necessary activities are outlined and clear, and implementation is where EDI work is challenged. The authors do make note of this in their discussion but could have pushed this further in what additional insights are necessary to help similar organizations embed EDI strategies as part of their remit.

10. **References** - are they up to date and relevant? Any

omissions? See previous comments on lack of global

11. **Abstract/summary/key messages** - do they reflect accurately what the paper says?

Ensure that the abstract and narrative of manuscript aligns.

12. **Reporting standards** - does the paper comply with relevant standards? If the paper is a randomised controlled trial we will have asked the authors to provide the protocol and a CONSORT checklist. Other research designs should have the relevant checklist included (PRISMA, STARD, etc).

SRQR reporting checklist used.

Other:

Simplify statements to omit unnecessary language to overstate the work. For example: page 4, line 84 – remove "unprecedented study" and simply use "study". Similarly, line 285,
p. 18 – "theoretically novel logic models" – I'm not certain what this means as I don't see what theory is extended through this work.

Glossary of abbreviations – for example, BRC, CRF, and EDI are all described but PPIE (line 195, p. 15 is not prior to its use – the list of abbreviations also is missing this line 475, p.28; MOU is also missing).

The abstract states that this is a pilot – but this isn't mentioned again. Please revise.

VERSION 1 - AUTHOR RESPONSE

**REVIEWER ONE**		
Very timely, very thorough - I like the paper very much. The graphics add much and I would just footnote them in terms of the sources for the data and the overall meaning so they can stand alone. Well done!	The figures have been revised and now include footnotes/captions explaining what they represent. Figures 4-6 in Supplementary data have also been edited to include footnotes.	Figures 1-3; Supplementary data
**REVIEWER TWO**		
Originality: This is an interesting topic and of importance to the research enterprise. While I appreciate the work is located within the UK – I would encourage the authors to expand the relevance of this work and describe additional work that may have been done globally that explore EDI frameworks and their outcomes, within research enterprises in similar health systems. This could easily be added to the	I have embedded the following paragraph into the introduction, prior to describing the EDI strategies across NIHR BRCs and CRFs. This paragraph provides context on the global relevance of the topic:<br><br>Internationally, research funders have developed structured EDI frameworks that link measurable progress to research excellence. For example, the Canadian Institutes of Health Research (CIHR) and its Tri-Agency partners (NSERC and SSHRC) have implemented a national EDI Action Plan that integrates inclusive participation, data transparency and accountability within research funding and	Line 9-20, Page 1

background and then referred again within the discussion.<br><br>For example, the Canadian Institutes of Health Research has an EDI strategy and framework where progress is reported. See: Equity, Diversity and Inclusion (EDI) in Action at CIHR - CIHR (see page 2, lines 49-50, rationale for work).<br><br>See also: Fonds de recherche du Québec (FRQ) Innovative-EDI-Practices-by-Granting-Agencies.pdf which describes EDI practices using case examples.	governance. Similarly, the Fonds de recherche du Québec (FRQ) has produced the Innovative EDI Practices by Granting Agencies framework, which documents concrete case examples of how granting agencies have reformed peer-review processes, broadened eligibility criteria and incorporated EDI indicators into evaluation and monitoring systems. Such international examples highlight the growing emphasis on embedding EDI at every stage of the research lifecycle, from funding and leadership to research participation. They also provide a useful benchmark for assessing the strategic maturity of NIHR-funded infrastructures such as BRCs and CRFs.	
Importance of work to readers: As previously stated, this work is timely and important to the research enterprise and decision-makers as the research community aims to embed EDI within their practices. See previous above to expand relevance of the work to a more global audience and situate this work within a global perspective – as written, including within the discussion, it is very focused on NIHR and relevance within the UK.	I have added the following statement to the Discussion (Implications) to reinforce that this study provides a transferable framework for other health research systems internationally:<br><br>While the present study focuses on NIHR-funded BRCs and CRFs in England, it offers insights that are transferable to other national research systems seeking to operationalise and evaluate EDI frameworks within multi-institutional infrastructures. Together, these international models (e.g. CIHR EDI) and the NIHR EDI frameworks highlight a global movement towards funder-led accountability and data-driven inclusion in health research.	Line 555-9, Page 24-25
Study design: While the research question(s) are provided, I have some comments on these. Design is appropriate to address at least the first two research objectives: 1) What are the recurring categories and priorities outlined within EDI strategies for both NIHR BRCs and CRFs? and 2) What are the similarities and differences in the stated objectives, action plans, and	While the design of this study, based solely on publicly available EDI strategy documents, did not permit empirically evaluative recommendations, the inductive content analysis nonetheless identified consistent conceptual gaps and areas for improvement across NIHR infrastructures, based on the EDI strategies.<br>Below are some areas of improvement that were identified and described in the discussion:<br>• Improvement needed in developing consistent, standardised frameworks across BRCs/CRFs,	Discussion: Line 475-498, Page 21-2<br><br>Research question: Line 108-10, Page 5

success measures within the EDI strategies of NIHR BRCs and CRFs? I am unclear about objective 3 in relation to the design: "**What recommendations can be made to enhance the strategic development, measurability and accountability of EDI strategies within NIHR-funded research infrastructure?**" (p.4, lines 95 to 97); whether the design of the study allows for this objective to be measured. The authors note that they have only relied on publicly available documents for analyses and a limitation is the lack of partner engagement to understand the nuances and implementation considerations surrounding these strategies. Unless these documents specifically address recommendations within their evaluation(s), the ability to answer this objective would be limited within current approach/project scope.	with clearer differentiation between planning (actions) and evaluation (measures).<br>• Improvement needed in defining measurable outcomes such as diversity metrics, training uptake, or recruitment improvements.<br>• Improvement needed in embedding governance accountability mechanisms and structured reporting systems across infrastructures.<br><br>I have adapted Research Question 3 so it now reflects that I am analysing what the documents themselves reveal about the clarity measurability and accountability of future EDI strategies:<br>What opportunities for strengthening the clarity, measurability and accountability of EDI strategies can be identified through a qualitative analysis of objectives, action plans and success measures across BRCs and CRFs?	
Methods - The authors situate this work within a constructivist paradigm using inductive content analyses. While the methods are appropriate, please add additional detail on ICA for the reader and supporting citations. Constructivist paradigm should also be referenced. (see p. 10, lines 100-104).	I have added the following description of ICA (under 'Data analysis') for readers who may be unfamiliar with the method:<br><br>ICA was chosen because it enables the systematic organisation and interpretation of textual data when there is limited prior research or theoretical guidance on the phenomenon under study (Elo & Kyngäs, 2008; Kyngäs, 2020). ICA involves open coding, categorisation, and abstraction to derive patterns and categories directly from the data rather than applying predefined frameworks. The method aligns with a constructivist paradigm, which assumes that knowledge and meaning are co-constructed through interpretation and context rather than discovered as objective	Line 171-9, Page 8

	truths (Lincoln & Guba, 1985; Charmaz, 2014). In this study, the constructivist stance guided the analytic process by recognising that the EDI strategies were developed within social and organisational contexts, reflecting institutional understandings of equality and inclusion within the NIHR research ecosystem.	
This research used publicly available documents; no human ethics required. However, did this study undergo an ethics exemption within the sponsoring institution? Please confirm and state the same or provide proof of exemption (will defer to BMJ editor for this). Please confirm that data management guidelines were followed based on NIHR Bristol Biomedical Research Centre institutional guidelines.	I confirm that this study received an ethics exemption because it involved analysis of publicly available, anonymised documents. As no human participants were involved and no identifiable or confidential information was collected, the work was classified as a secondary analysis of publicly available data and did not require formal ethics committee approval. All data management procedures adhered to the NIHR Bristol Biomedical Research Centre's institutional data governance and security guidelines, including secure storage, version control, and restricted access to project files.<br><br>I have added the following statement under 'Ethical considerations':<br>This study received an ethics exemption, as it involved secondary analysis of publicly available, anonymised documents. No human participants or confidential information were included. Accordingly, formal ethics approval was not required. All data were managed and stored in compliance with institutional data governance and security policies of the NIHR Bristol Biomedical Research Centre.	Line 126-30, Page 6
Search and Analysis: p. 11 – lines 125-27 – who is we? Were websites searched by 1 author or multiple authors?<br><br>Was a search approach used for website search? For example, how many webpages were followed? Were Boolean operators and or specific phrases applied to each webpage? What was searched for? More details on the systematic approach would be helpful.	I have now clarified on Page 8 that 'We' refers to the lead author (under the supervision of the co-authors).<br><br>I confirm that a systematic approach was used to identify all publicly available EDI strategy documents. The search involved visiting the official websites of each of the 20 NIHR BRCs and 28 CRFs individually, using the most recent version of the EDI strategy provided on the NIHR website. Within each site, the internal search function and navigation menus were used to locate pages or documents labelled with terms such as "equality, diversity and	Line 180, Page 8<br><br>Line 139-144, Page 7

	inclusion," "EDI strategy," "EDI action plan," "research inclusion," or "EDI report." Boolean operators were not required as all websites were searched manually but the same search terms were applied consistently to ensure comparability. All documents meeting these criteria were downloaded and stored in PDF format for analysis.<br><br>I have edited the paragraph under 'Data collection and materials' to clarify the systematic search approach:<br><br>The lead researcher conducted a systematic search in June 2024 to identify all publicly available EDI strategy documents for NIHR BRCs and CRFs. The official websites of all 20 BRCs and 28 CRFs listed on the NIHR website were searched manually using site search functions and navigation menus. Consistent keywords were applied across sites, including Equality, Diversity and Inclusion, EDI strategy, EDI action plan, research inclusion, and EDI report. All eligible documents were downloaded and archived in PDF format.	
Further, if there were multiple reviewers, was there a pilot and then an IRR conducted? Please explain. Were there specific inclusion/exclusion criteria? Mention this. There are a noted lack of details in the methods.	The following paragraph has been added to 'Data analysis':<br><br>In this study, because the analysis was conducted within a constructivist paradigm, we did not calculate formal inter-rater reliability statistics. Instead, we used the qualitative approach of iterative, consensus-based coding to ensure analytic rigour. The lead author conducted initial open coding on a subset of EDI strategies (e.g., 10% of the total documents) to develop a preliminary coding framework. This framework, along with coded extracts, was reviewed by the co-authors. Differences in interpretation were discussed collaboratively until consensus was reached and the coding framework was refined through multiple iterations. The iterative process of coding, discussion and refinement of the coding framework continued until all documents were coded and no new categories	Line 182-192, Pages 8-9

	emerged, ensuring saturation of themes. This process facilitated shared understanding and consistency across coders while remaining aligned with the principles of inductive content analysis.<br><br>The following paragraph has been added to 'Data Collection and materials' to clarify inclusion/exclusion criteria:<br><br>Regarding inclusion and exclusion criteria, our dataset comprised all publicly available NIHR BRC and CRF EDI strategy documents in England. Documents were included if they met the following criteria:<br>(1) explicitly identified as an EDI strategy, action plan or comprehensive EDI report;<br>(2) publicly accessible on an official NIHR BRC/CRF or affiliated institutional website.<br>Documents were excluded if they:<br>(1) were internal-only, non-public or operational documents not intended as formal EDI strategies;<br>(2) focused on equality legislation compliance rather than strategic EDI objectives; or<br>(3) were brief statements without substantive strategy content.	
Line 127 – the sentence that starts with "some were written" – please clarify what "some" is – does this refer to the strategies? Lines 128 to 130 also read like findings.	I have edited this statement to clarify that 'some' refers to the 14 BRCs with the same EDI strategy as their corresponding CRFs.<br><br>Fourteen BRCs had the same EDI strategy as their corresponding CRFs and 16 CRFs had separated EDI strategies…	Line 155-6, Page 7
P. 12 – lines 147 to 149 – how was reliability of coding assessed among multiple reviewers?<br>Or was there only one coder for all documents? Please clarify.	I have added this paragraph to the 'Data analysis' section:<br><br>In this study, because the analysis was conducted within a constructivist paradigm, we did not calculate formal inter-rater reliability statistics. Instead, we used the qualitative approach of iterative, consensus-based coding to ensure analytic rigour. The lead author conducted initial open coding on a subset of EDI strategies to develop a preliminary coding framework. This framework, along with coded extracts, was reviewed by the co-authors. Differences in interpretation were discussed collaboratively until consensus	Line 182-192, Pages 8-9

	was reached and the coding framework was refined through multiple iterations. This process ensured shared understanding and consistency across coders while remaining aligned with the principles of inductive content analysis.	
There seem to be some inconsistencies with what is written in the methods, and the detail/information provided on the SRQR and in the abstract. The comments made in relation to the SRQR should be embedded within the narrative of the manuscript. For example, Excel is mentioned as an organization tool for the analysis (also in abstract) but how it's used is not described within the methods section.	To address the discrepancy between the SRQR and manuscript, I have added the following statement under 'Data analysis' to explain how data were processed after being imported into Nvivo:<br><br>In addition to NVivo, Microsoft Excel was used to organise and manage the descriptive elements of the analysis. Excel spreadsheets were developed to catalogue each strategy document, track the presence or absence of codes across BRCs and CRFs and calculate the frequency of sub- and main categories generated during coding. These spreadsheets also allowed comparisons to be mapped and systematic examination of similarities and differences across infrastructures.	Line 163-8, Page 8
Results - The authors organize their findings narratively and in table format, with logic models developed. Would the authors consider organizing their findings in alignment with their study objectives? This would help with reader interpretation.	Our first two study aims were:<br>(1) identify recurring categories and priorities within EDI strategies across all 20 NIHR BRCs and the 28 CRFs, and<br>(2) compare similarities and differences in how these categories were represented across objectives, action plans and success measures.<br>I have now asserted these aims under 'Aims and research questions'.<br><br>Under 'Qualitative content analysis of EDI strategies BRCs/CRFs', I address Research Question 1 by summarising the recurring categories for Objectives, Action Plans and Success Measures (e.g. Evaluations/Audits, Student/Career Development etc.).<br>Under each category, I then explain similarities and differences between BRCs and CRFs in terms of how they integrated each of these categories into their EDI strategies.<br><br>I have decided to adapt Research Question 2 so that it more accurately reflects the aims of the study and the findings I	Aims: Line 96-8, Page 4<br><br>Qualitative content analysis: Line 215-324, Page 11-15<br><br>Research question 2: Line 105-7, Page 5

	subsequently summarised: What categories or priorities do NIHR BRC and CRF EDI strategies articulate across objectives, action plans and success measures? What are the similarities and differences in these categories/priorities across BRCs and CRFs?<br>I address this research question under 'Common main categories across objectives, action plans and success measures for BRCs/CRFs'. I also address similarities and differences between the two infrastructures (e.g. BRCs generally had higher thematic prevalence; CRFs had less developed EDI strategies).<br><br>The approach I have taken of summarising categories is also consistent with qualitative content analysis, where results are organised around the categories that emerge from the data rather than the analytic questions that guided data collection.	
At this stage, and with the evidence they have collected, I don't know if the separate logic models contribute anything further to the findings and detract from the purpose of this work. Why have separate logic models for each category?	The logic models were developed to translate the written summary of categories into a practical and accessible visual tool for stakeholders designing, implementing and evaluating EDI strategies within NIHR infrastructures. We intended not only to describe patterns within existing strategies, but also to support NIHR Inclusion Leads, governance teams and research managers to reflect on how objectives, action plans and success measures can be more systematically aligned. The logic models, therefore serve as a succinct visual representation of this alignment, offering an infographic-style resource that stakeholders can use to guide planning and stimulate discussion around their own strategies.<br><br>Further, these models were originally created for the inaugural national NIHR BRC/CRF Inclusion Conference, where they were used to facilitate dialogue among EDI leads and senior research leaders. Feedback from NIHR Inclusion Managers, BRC/CRF EDI Leads and academic colleagues expressed that the logic models filled a recognised gap by synthesising	Figures 1-3, Supplementary data

	complex findings into an actionable format. Several stakeholders requested that the models be shared more widely to support future strategy development. In light of this feedback, we felt it was important to include the logic models in the manuscript as an academic contribution and practical resource.	
Pages 13 to 14 describe well known approaches to conducting planning and evaluation work within service-oriented organizations and do not provide novel contributions to the literature. In another example, success measures are presented as outputs rather than evaluation metrics – e.g., toolkits, communications plans, and webpages.	I agree that concepts such as research development, collaborations and governance are widely recognised within organisational planning and evaluation. However, the contribution of this analysis lies not in naming these concepts, but in demonstrating systematically and for the first time, how they are articulated and operationalised across all NIHR BRC and CRF EDI strategies.<br>This article also provides an pilot mapping of how each category spans objectives, action plans and success measures, including their depth and maturity across BRCs and CRFs.<br><br>I agree that some success measures reported in the strategies (e.g., toolkits, communication plans, webpages) reflect outputs rather than evaluative indicators. This is one of the key findings of the study. This analysis highlights a tendency across infrastructures to name deliverables as indicators of strategic progress and success (rather than evaluation metrics). I have included a statement about this under the Discussion:<br><br>Some success measures reported in the strategies (e.g., toolkits, communication plans, webpages) reflect outputs rather than evaluative indicators. This signifies a tendency across BRCs/CRFs to name deliverables as indicators of strategic progress and success (rather than evaluation metrics).	Line 429, Page 19
Interpretation and conclusions - The message isn't as clear as it could be and I think this has to do with the organization of the findings/results as well as the depth of reflections found	I've added the following statement to the Discussion to clarify the main messages:<br><br>We acknowledge that concepts identified in our main categories, such as research development, collaborations and governance, research development,	Line 514-520, Page 23

within the discussion section.<br><br>The authors should be commended for conducting this work – there is a clear need to understand how research-supporting entities like BRCs and CRFs can integrate EDI into their practices.	collaborations and governance are widely recognised within organisational planning and evaluation. However, the contribution of our analysis lies not in naming these concepts, but in demonstrating systematically and for the first time, how they are articulated and operationalised across all NIHR BRC and CRF EDI strategies. This article also provides a pilot mapping of how each category spans objectives, action plans and success measures, and their depth and maturity across BRCs and CRFs.<br><br>The emergence of six cross-cutting categories across objectives, action plans and success measures, and the differential strategic maturity between BRCs and CRFs, is already foregrounded in the opening paragraph of the Discussion.	
While the authors do discuss implementation in their discussion, I would encourage them to consider further the limitations of what they have observed (see: lines 361-363, lack of detail on what's been achieved and why some strategies may or may not be successful). Are we at risk of finding success in low-hanging strategies where the more important strategies – governance, leadership, policy may be more impactful? Moreover, are there strategies that they didn't observe, that the literature suggests could be important?	The Discussion section already describes limitations of the findings such as:<br>• There is variability in strategy depth, implementation planning and evaluation metrics and further development is required to achieve long-term, measurable progress.<br>• There could also be efforts to differentiate between how action plans and success measures are outlined in EDI strategies.<br>• To deploy these EDI strategies, there continues to be heavy reliance on the NIHR workforce and increased involvement of research participants and the public could maximise inclusion.<br>• We cannot determine from these EDI strategies, what impacts success measures have had and whether they were achieved.<br><br>I have added the following points to the Discussion which acknowledge further limitations of the findings:<br>• Although the strategy documents provide insight into objectives, action plans and intended success measures, they do not reveal whether initiatives have been implemented as planned, nor	Line 475-498, Page 21-2<br><br>Line 530-534, Page 23-4<br><br>Line 537-541, Page 24

	whether they have led to measurable changes. Further comparative evaluations of EDI strategies could assess the effectiveness or maturity of implementation.<br>• We address the possibility that there are widely known strategies or approaches (e.g., systems-level equity frameworks, equity-centred evaluation models, organisational change frameworks, structural accountability mechanisms) that were not consistently observed across BRCs and CRFs. Their absence in the data does not imply that they are not being used within NIHR infrastructures, only that they were not present in publicised strategies.	
I'm also focusing on the authors use of "embedded" in relation to the identified strategies (p. 12, lines 161-162). My interpretation of the aim of this work is to categorize the EDI strategies as they've been named or identified within these NIHR entities – linking to objective 1, and to describe similarities and differences between the BRCs and CRFs – addressing objective 2.<br><br>From the detail provided further along in the authors' analysis, the existing level of operationalization or "embeddedness" is open to interpretation. For example, evidence of evaluation findings varies across the documents reviewed and how success is defined varies according to the strategy and/or action. Based on this – is there even an understanding of the progress made on each of these actions/objectives?	I agree that publicly available strategy documents do not provide sufficient evidence to assess whether EDI commitments have been implemented, operationalised or embedded within organisational practice. This study did not aim to evaluate effectiveness or progress, nor did we seek to determine whether strategies had been implemented as written. This analysis focused on how EDI priorities are presented and articulated, not how they are executed. I have added a section under 'Implications for NIHR and EDI leads and stakeholders' to reinforce that this article does not provide an understanding of progress made on each of the actions/objectives:<br><br>This study was based solely on publicly available strategy documents which did not provide an assessment of the progress made on individual actions or objectives. Likewise, we could not determine the extent to which any strategy had been implemented in practice. It is also notable that the term 'embedded' was used within the EDI strategies to describe intentions or aspirations, yet the documents did not provide sufficient detail to evaluate whether such aspirations had translated into operational change. We additionally	Line 542-554, Page 24

Of note is that some outputs are named but few details provided. I'm not clear that the evidence within this study can speak (at this point) to the level of embeddedness of EDI strategies or not. This is one of the inherent challenges of EDI work – it is well-meaning, needed/necessary activities are outlined and clear, and implementation is where EDI work is challenged. The authors do make note of this in their discussion but could have pushed this further in what additional insights are necessary to help similar organizations embed EDI strategies as part of their remit.	acknowledge that this term appeared in our coding framework as part of the naming of subcategories. Taking into account the reviewer's feedback, this terminology was not the most appropriate descriptor for what could be evidenced from the data, and we recognise this as a limitation of the analysis. Future research would be required to understand implementation and progress, including approaches such as interviews with EDI leads, case studies of how strategies are operationalised and evaluation using agreed indicators. These studies can provide a greater understanding of whether and how EDI strategies are truly 'embedded' within NIHR infrastructures.<br><br>I have revised the manuscript to reduce use of the term 'embedded' except when this is the exact term used in the EDI strategy.	
Abstract - Ensure that the abstract and narrative of manuscript aligns.	I have revised the Conclusions of the Abstract to ensure it aligns with the narrative of the manuscript:<br><br>This study provides the first systematic analysis of EDI strategies across all NIHR BRCs and CRFs, offering a comprehensive mapping of how EDI priorities are articulated and operationalised across objectives, action plans and success measures. While both infrastructures align with NIHR's inclusion goals, BRCs generally showed more strategic maturity than CRFs. As the analysis was based solely on publicly available strategy documents, it could not determine the extent to which any strategy had been implemented in practice. Future research is needed to examine implementation and impact. The contribution of this work lies in demonstrating systematically and for the first time, the ways in which EDI commitments are framed across NIHR infrastructures and their varying levels of depth and maturity. Our findings support the development of more measurable EDI	'Conclusions' under Abstract

	frameworks and highlights opportunities to strengthen inclusion across NIHR-funded research infrastructures.	
Other - Simplify statements to omit unnecessary language to overstate the work. For example: page 4, line 84 – remove "unprecedented study" and simply use "study". Similarly, line 285, p. 18 – "theoretically novel logic models" – I'm not certain what this means as I don't see what theory is extended through this work.	• I have removed "unprecedented study" from Page 4<br>• I have removed "theoretically novel" from Page 16 and simply stated that they are "novel logic models"	Line 96, Page 4<br>Line 347, Page 16
Glossary of abbreviations – for example, BRC, CRF, and EDI are all described but PPIE (line 195, p. 15 is not prior to its use – the list of abbreviations also is missing this line 475, p.28; MOU is also missing).	• I have now added PPIE to the Glossary of Abbreviations and spelled out the acronym in full the first time it is used.<br>• I have now added MOU to the Glossary of Abbreviations.	Page 26
The abstract states that this is a pilot – but this isn't mentioned again. Please revise.	I have retained use of the word 'pilot' to describe the nature of the analysis in the abstract. For consistency, I have reinforced the pilot nature of the study's analysis throughout the manuscript including the Aims, Methods and Discussion. I hope these revisions provide clearer alignment between the abstract and the main text regarding the pilot nature of the study.	Abstract<br>Line 170, Page 8<br>Line 384, Page 18<br>Line 519, Page 23<br>Line 561, Page 25

VERSION 2 - REVIEW

**Reviewer**	**2**
**Name**	**Sim, Meaghan**
**Affiliation**	**IWK Health Centre**
**Date**	**30-Dec-2025**
**COI**	

Thank you for the opportunity to review this revised manuscript. The authors' attendance to revisions is noted and improves the manuscript.
